# The Anti-SLAMF7 Antibody, Elotuzumab, Induces Antibody-Dependent Cellular Cytotoxicity Against CLL Cell Lines

**DOI:** 10.3390/molecules31030531

**Published:** 2026-02-03

**Authors:** Dominik Kľoc, Bianca Dubiková, Simona Žiláková, Ján Sykora, Michaela Šuliková, Slavomír Kurhajec, Ján Sabo, Tomáš Guman, Marek Šarišský

**Affiliations:** 1 Department of Pharmacology, Faculty of Medicine, Pavol Jozef Šafárik University in Košice, Trieda SNP 1, 04011 Košice, Slovakia; dominik.kloc@student.upjs.sk (D.K.); bianca.dubikova@student.upjs.sk (B.D.); simona.zilakova@student.upjs.sk (S.Ž.); 2 Department of Haematology and Oncohaematology, Faculty of Medicine, Pavol Jozef Šafárik University in Košice and Louis Pasteur University Hospital Košice, Trieda SNP 1, 04011 Košice, Slovakia; jan.sykora@unlp.sk (J.S.); tomas.guman@upjs.sk (T.G.); 3 Department of Medical and Clinical Biophysics, Faculty of Medicine, Pavol Jozef Šafárik University in Košice, Trieda SNP 1, 04011 Košice, Slovakia; michaela.sulikova@upjs.sk (M.Š.); jan.sabo@upjs.sk (J.S.); 4 Department of Pharmaceutical Technology, Pharmacognosy, and Botany, University of Veterinary Medicine and Pharmacy, Komenského 73, 04181 Košice, Slovakia; slavomir.kurhajec@uvlf.sk

**Keywords:** chronic lymphocytic leukemia, CLL, SLAM family receptors, SLAMF, antibody-dependent cellular cytotoxicity, ADCC, elotuzumab, rituximab, flow cytometry, Mec-1

## Abstract

SLAMF7, also known as CD319, a SLAM (signaling lymphocytic activation molecule) family receptor, is relatively weakly expressed on chronic lymphocytic leukemia (CLL) B cells. This study evaluated the ability of elotuzumab (E), an anti-SLAMF7/CD319 antibody, to induce antibody-dependent cellular cytotoxicity (ADCC) against CLL cell lines (MEC-1, MEC-2, CI, HG-3, PGA-1, WA-OSEL). ADCC was assessed by flow cytometry using E (100 μg/mL), rituximab (R, 100 μg/mL), and their combination (E + R). CLL lines served as targets (T), while peripheral blood mononuclear cells (PBMCs) or NK cells from healthy donors served as effectors (E) at an 8:1 E:T ratio for 4 h. With PBMCs, E-induced ADCC ranged from 1.3 ± 1.2% (PGA-1) to 14.6 ± 8.1% (MEC-1); R-induced ADCC ranged from 9.2 ± 4.6% (PGA-1) to 16.6 ± 9.4% (WA-OSEL). With NK cells, E-induced ADCC ranged from 1.8 ± 3.7% (PGA-1) to 27.3 ± 4.7% (MEC-1); R-induced ADCC ranged from 5.1 ± 4.3% (PGA-1) to 27.5 ± 13.6% (CI). E outperformed R in MEC-1, while R was superior elsewhere. Cell lines with higher SLAMF7/CD319 expression displayed increased sensitivity to E. Cell lines with del17p showed higher SLAMF7/CD319 expression. The combination of E + R showed no significant synergy over monotherapies. In conclusion, elotuzumab induced significant ADCC in CLL cells, warranting further therapeutic evaluation.

## 1. Introduction

Chronic lymphocytic leukemia (CLL) is a hematological malignancy characterized by the presence of ≥5 × 10^9^/L neoplastic mature B cells in the peripheral blood [1,2] that show a characteristic immunophenotypic profile (in particular, CD5 and CD23 positivity with weak or negative expression of CD20, CD79b, and IgM) and immunoglobulin light chain restriction demonstrated by flow cytometry [3,4]. The incidence of CLL in Western countries typically ranges from 3 to 5 cases per 100,000 people per year. For example, in the United States, the age-adjusted incidence rate of CLL is approximately 4.7 per 100,000 per year [5]. In Western countries, CLL represents the most common type of leukemia in adults, accounting for approximately 25–30% of all diagnosed leukemia cases [6]. Different targeted therapies are available for the treatment of CLL: (i) Bruton tyrosine kinase inhibitors (BTKis) (ibrutinib, acalabrutinib, zanubrutinib), (ii) B-cell lymphoma 2 inhibitor (BCL2i) (venetoclax), (iii) phosphoinositide 3-kinase (PI3K) inhibitor (idelalisib), and (iv) anti-CD20 antibodies (obinutuzumab, rituximab) [7]. Despite the continued development of novel therapeutic strategies, CLL remains an incurable disease. Therefore, the identification of new therapeutic targets and the development of novel, more effective, targeted therapies for CLL are of great importance.

The signaling lymphocytic activation molecule (SLAM) receptor family (SLAMF) consists of nine glycoproteins that belong to the CD2 superfamily of immunoglobulin (Ig) domain-containing molecules. SLAMF receptors are expressed on hematopoietic cells and are recognized as important immunomodulatory receptors with roles in cytotoxicity, humoral immunity, autoimmunity, cell survival, lymphocyte development, and cell adhesion [8]. Among B-cell chronic lymphoproliferative disorders (B-CLPD), the expression of SLAMF receptors is best characterized in CLL [9]. In our previous study, we found that SLAMF1/CD150, SLAMF2/CD48, and SLAMF7/CD319 are downregulated while SLAMF3/CD229, SLAMF5/CD84, and SLAMF6/CD352 are overexpressed in CLL B cells in comparison with normal mature polyclonal B cells [10]. Alterations in the expression of these receptors may contribute to the aggressive course of CLL, therapeutic resistance, and immune dysfunction observed in affected patients. Compared to other SLAMF receptors, the expression of SLAMF7/CD319 is weak in CLL [10,11]. On the other hand, its expression in plasmablastic lymphoma (PBL) and primary effusion lymphoma (PEL) was found to be strong. In PBL, the expression of SLAMF7/CD319 was comparable to that in OPM2 multiple myeloma cells [12], while its expression in PEL cell lines was stronger than in myeloma cell lines [13]. SLAMF7/CD319 positivity was also observed in Burkitt lymphoma cell lines [14]. In contrast, Z138 mantle lymphoma cells were found to be SLAMF7/CD319-negative [13]. The expression of SLAMF7/CD319 in other B-CLPD subtypes has not yet been characterized.

Antibodies targeting multiple SLAMF receptors have been tested for their potential in the treatment of CLL. These include antibodies against SLAMF1/CD150 [15], SLAMF2/CD48 [16], SLAMF5/CD84 [17], and SLAMF6/CD352 [11,18,19]. The effect of anti-SLAMF7/CD319 antibodies against CLL cells has not yet been studied, but it was demonstrated that elotuzumab, a therapeutic anti-SLAMF7/CD319 monoclonal antibody approved for the treatment of relapsed/refractory multiple myeloma, is capable of inducing antibody-dependent cellular cytotoxity (ADCC) against SLAMF7/CD319-positive PBL [12] and PEL cells [13].

In the present work, our goal was to analyze the ability of elotuzumab to induce ADCC against a panel of six CLL cell lines and to assess its potential in the treatment of CLL.

## 2. Results

### 2.1. Immunophenotypic Characteristics of CLL Cell Lines

All CLL cell lines used in this study were first immunophenotypically characterized by flow cytometry using the adapted EuroFlow B-CLPD panel. All cell lines expressed CD19, CD20, and CD45. MEC-1, MEC-2, and WA-OSEL cells showed a monotypic expression of immunoglobulin kappa light chains, while CI, HG-3, and PGA-1 expressed immunoglobulin lambda light chains. The antigen CD5 was strongly positive in CI and HG-3, negative to dimly positive in PGA-1, and completely negative in MEC-1, MEC-2, and WA-OSEL. In the case of HG-3, CD5 expression was bimodal, with approximately 40% negative cells and 60% brightly positive cells. The strong expression of CD23 was observed in all cell lines. The expression of CD22 was intermediate (CI and HG-3) to strong (MEC-1, MEC-2, and WA-OSEL). The antigen CD79b was clearly positive in MEC-1 and WA-OSEL, dimly positive in MEC-2, HG-3, and PGA-1, but absent in CI. Cell surface IgM was present in all cell lines except PGA-1. All cell lines showed weak to intermediate levels of CD200 expression. These immunophenotypic characteristics are principally in line with a phenotype typical for CLL (CD5+, CD23+, CD22−/weak, CD79b−/weak, IgM−/weak, CD200+). Regarding the remaining antigens, all cell lines showed a strong to very strong expression of HLA-DR, CD39, CD43, CD49d, and CD81. Antigens CD27, CD38, and CD62L displayed heterogeneous expression among cell lines. Finally, CD31, CD95, CD185, and CD305 were negative to dimly positive, while CD10, CD11c, and CD103 were consistently negative in all cell lines (see Supplementary Figure S1).

**Figure 1:**
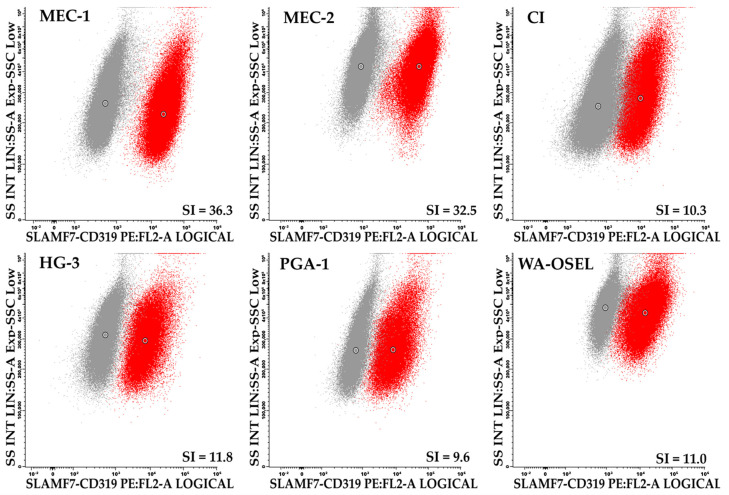
Expression of the SLAMF7 receptor in CLL cell lines. Grey dots, control unstained cells; red dots, cells stained with the indicated antibodies.

Regarding the expression of SLAMF receptors, SLAMF7/CD319 was positive in all CLL cell lines. Its expression was particularly strong in MEC-1 and MEC-2 cells, while the remaining CLL cell lines showed approximately three times lower levels of expression in terms of the stain index (SI) (Figure 1).

Overall, the most intensely expressed SLAMF receptor was SLAMF6/CD352 (SI = 16.4–54.4, range for all six cell lines) and SLAMF1/CD150 (SI = 24.0–50.1), followed by SLAMF7/CD319 (SI = 10.3–36.3). Intermediate levels of expression were observed for SLAMF2/CD48 (SI = 6.8–15.3) and SLAMF3/CD229 (SI = 4.4–9.9), while SLAMF5/CD84 was expressed only relatively weakly (SI = 1.0–8.5). The SLAMF9/CD84 receptor was weakly expressed in MEC-1 cells (SI = 1.9), very weakly expressed in MEC-2 (SI = 0.9) and WA-OSEL (SI = 0.7) cells, and absent in the remaining CLL cell lines (SI = 0.3–0.6). The expression of intracellular SLAM-associated adaptor proteins SAP and EAT-2 was of intermediate intensity: SI = 3.5–8.6 and SI = 2.1–28.6, respectively. Finally, all CLL cell lines were negative for SLAMF4/CD244 (SI = 0.2–0.8) and SLAMF8/CD353 (SI = 0.2–0.5) (see Supplementary Figure S2).

### 2.2. Antibody-Dependent Cellular Cytotoxicity

**Figure 2:**
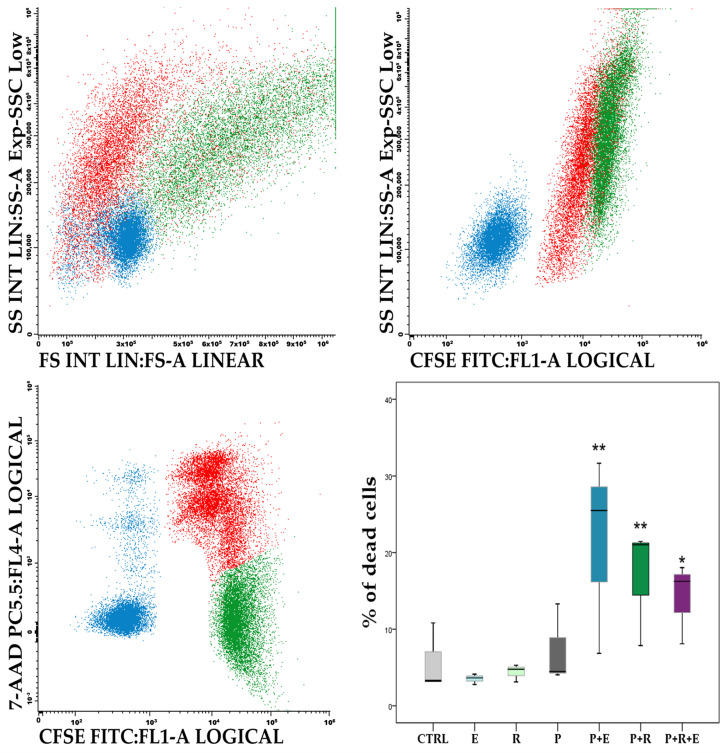
The analysis of ADCC in MEC-1 cells. Illustrative bivariate dotplots (FSC/SSC, CFSE/SSC, and CFSE/7-AAD) showing live target (T) MEC-1 cells (green, CFSE+, 7-AAD−), dead target (T) MEC-1 cells (red, CFSE+, 7-AAD+), and live and dead effector (E) PBMCs (blue, CFSE−, 7-AAD− or +). The graph (lower right) shows the percentage of dead cells in control (CTRL) MEC-1 cell culture and MEC-1 cells incubated for 4 h with elotuzumab (100 μg/mL) alone (E), rituximab (100 μg/mL) alone (R), PBMCs alone at an 8:1 (E:T) ratio (P), PBMCs and elotuzumab (P + E), PBMCs and rituximab (P + R), and PBMCs and elotuzumab/rituximab combination (P + R + E). Statistical significance * *p* < 0.05, ** *p* < 0.01.

Using a standard 4 h flow cytometric ADCC assay with PBMCs as effector cells, we found that elotuzumab (100 μg/mL) was able to induce significant ADCC (mean %ADCC ranging from 4.2% to 14.6%) in four out of six CLL cell lines used, namely Mec-1 (Figure 2), Mec-2, HG-3, and WA-OSEL (Table 1). On the other hand, rituximab (100 μg/mL), which was used as a positive control, induced significant ADCC (mean %ADCC ranging from 9.2% to 18.0%) in all CLL cell lines, including CI and PGA-1, which were insensitive to elotuzumab. Prolongation of the incubation time to 24 h did not lead to increased ADCC.

When unstimulated, untouched NK cells were used as effector cells, significant levels of elotuzumab-induced ADCC were observed in MEC-1, MEC-2, and CI cells (mean %ADCC ranging from 13.7% to 27.3%), while rituximab induced significant ADCC in MEC-2, CI, HG-3, and WA-OSEL cells (mean %ADCC ranging from 18.4% to 27.5%). NK cells were significantly more potent than PBMCs in inducing ADCC in MEC-1 (mean %ADCC of 14.6% vs. 27.3%, *p* < 0.05) and CI (mean %ADCC of 1.8% vs. 17.2%, *p* < 0.05) cells, while in the remaining cell lines, these differences were also observed, but they did not reach statistical significance.

Rituximab was a more potent ADCC-inducer than elotuzumab in all CLL cell lines except MEC-1, in which elotuzumab was more potent than rituximab (mean %ADCC of 14.6% vs. 12.1% with PBMCs and 27.3% vs. 22.5% with NK cells as effectors). PGA-1 cells were found to be the least sensitive of all cell lines to elotuzumab and rituximab (<2% and <10% of ADCC, respectively). Surprisingly, the addition of elotuzumab to rituximab did not lead to any synergistic effect; elotuzumab did not enhance the effect of rituximab regardless of the type of effector cells used.

**Figure 3:**
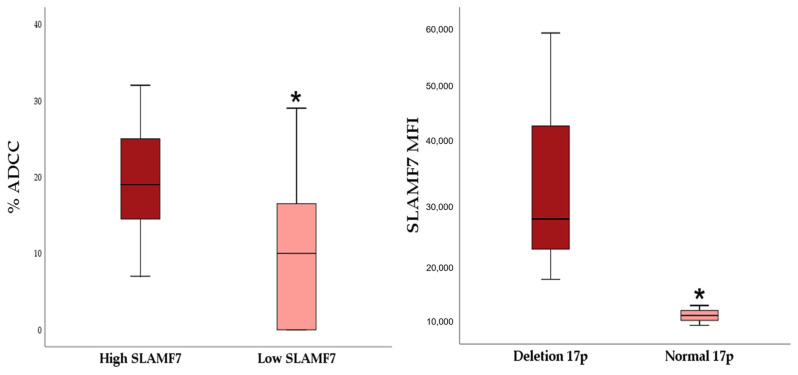
Relationships observed between SLAMF7/CD319 expression and %ADCC and the presence of 17p deletion. CLL cell lines that showed higher levels of SLAMF7/CD319 expression (MEC-1 and MEC-2) were more sensitive to elotuzumab-induced ADCC than cell lines with lower expression (CI, HG-3, PGA-1, WA-OSEL) (**left**). CLL cell lines with deleted chromosome 17p (MEC-1, MEC-2, and WA-OSEL) showed a higher intensity of SLAMF7/CD319 expression than cell lines with normal chromosome 17p status (**right**). Statistical significance * *p* < 0.05.

The ability of elotuzumab to induce ADCC was significantly higher in those CLL cell lines that expressed higher levels of SLAMF7/CD319 (MEC-1, MEC-2, MEC-2, and WA-OSEL) in comparison with cell lines that showed lower expression (CI, HG-3, and PGA-1) (*p* < 0.05) (Figure 3). Interestingly, cell lines harboring the deletion of chromosome 17p (MEC-1, MEC-2, and WA-OSEL) showed significantly stronger SLAMF7/CD319 expression than cell lines with normal chromosome 17p status (CI, HG-3, PGA-1) (*p* < 0.05). However, no relationships were observed between ADCC and chromosome 17p status, IgHV mutational status, SAP expression, and the expression of EAT-2.

## 3. Discussion

In this study, we evaluated elotuzumab-mediated ADCC against CLL cell lines. Firstly, we comprehensively characterized the used CLL cell lines in terms of their B-CLPD immunophenotypic profile and their SLAMF receptor expression. The expression of markers such as CD19, CD20, CD5, CD10, CD23, CD38, HLA-DR, IgM, Igkappa, and Iglambda was in full agreement with previous findings [20,21]. Apart from these markers, we also analyzed the expression of additional antigens, including CD11c, CD22, CD27, CD31, CD39, CD43, CD62L, CD79b, CD81, CD95, CD103, CD185, CD200, and CD305, which are important for the diagnosis/differential diagnosis of B-CLPD. The expression patterns of CD11c, CD39, CD43, CD62L, CD79b, CD95, CD103, CD200, and CD305 expression were typical for CLL. On the other hand, the expression of CD22 and CD81 was higher, while the expression of CD27, CD31, and CD185 was lower than expected for a typical CLL [21]. Taken together, the immunophenotypic characteristics of all six CLL cell lines used in this study were generally compatible with the immunophenotype typical of CLL. It should be noted that MEC-1 and MEC-2 are cell lines derived from a patient with CLL in prolymphocytic transformation.

Regarding SLAMF receptors, CLL cell lines displayed a high intensity of SLAMF6/CD352, SLAMF1/CD150, and SLAMF7/CD319 expression, intermediate intensity of SLAMF2/CD48 and SLAMF3/CD229, and relatively weak expression of SLAMF5/CD84. Weak or very weak expression of SLAMF9/CD84-H1 was detected in MEC-1, MEC-2, and WA-OSEL cells. The remaining SLAMF receptors (SLAMF4/CD244 and SLAMF8/CD353) were negative in all CLL cell lines. Compared to our previous findings in CLL patients [10], the expression of three SLAMF receptors in CLL cell lines differs from that on B cells from patients with CLL: SLAMF1/CD150 and SLAMF7/CD319, which show a markedly stronger expression, while SLAMF5/CD84 displays a notably weaker expression. The observation of the weak expression of SLAMF9/CD84-H1 in some CLL cell lines, especially MEC-1, is our original finding, as it has not been reported yet in the literature. In the present study, intracellular SAP and EAT-2 were tested positive with weak to intermediate expression intensity. EAT-2 expression in CLL has been previously reported [22]. On the other hand, SAP expression is generally considered lacking in CLL and other B-CLPDs, except for Burkitt lymphoma and diffuse large B-cell lymphoma [23,24,25,26]. This discrepancy between our results and other studies could be due to the different methodologies used: flow cytometry versus immunohistochemistry or mRNA expression analysis.

The expression of SLAMF7/CD319 on CLL cell lines used in this study was of intermediate to strong intensity, which provides the basis for testing anti-SLAMF7/CD319 strategies as a potential therapy for CLL. We employed an adapted flow cytometric ADCC assay according to Yamashita [27] to evaluate the ability of elotuzumab, an anti-SLAMF7/CD319 antibody, to induce ADCC against CLL cells using PBMCs or immunomagnetically isolated NK cells (untouched and unstimulated) from the peripheral blood of healthy donors as effector cells. Based on the mechanism of action of elotuzumab, which involves not only ADCC and ADCP (antibody-dependent cellular phagocytosis) but also the direct activation of NK cells through its SLAMF7/CD319 receptors and enhancement of NK-tumor cell interaction via facilitation of SLAMF7/SLAMF7 ligation [28], we also hypothesized that elotuzumab could act synergistically with rituximab, anti-CD20 antibody, and potentiate its action.

We found that elotuzumab can induce significant ADCC against CLL cell lines (4.2% to 27.3% ADCC). The percentage of ADCC observed in our study is generally consistent with the results of other groups that reported ADCC in the range of approximately 20–40% when using myeloma cell lines (including U266, RPMI 8226, and MM.1S) or patient myeloma cells as target cells, NK-92 cells or expanded NK cells as effector cells, E:T ratios from 5:1 to 10:1, and elotuzumab at concentrations of 20–100 μg/mL. The maximum ADCC of about 75% was achieved when effector NK-92 cells were transduced with high-affinity FcγRIIIA variants [29,30,31]. The effect of elotuzumab on individual CLL cell lines seems to depend on their expression level of the SLAMF7/CD319 receptor. Several studies have demonstrated that the efficacy of monoclonal antibodies is largely dependent on the level of expression of their target antigen. For example, preclinical studies have shown that rituximab-mediated killing of CLL and lymphoma cells depends on CD20 expression [32,33,34]. Clinical studies have confirmed poorer outcomes in patients with low CD20 expression treated with rituximab-based immunochemotherapy [35,36]. In our study, CLL cell lines that exhibit higher levels of SLAMF7/CD319 expression (MEC-1 and MEC-2) were more sensitive to elotuzumab-induced ADCC than cell lines with lower SLAMF7/CD319 expression (CI, HG-3, PGA-1, and WA-OSEL). On the other hand, elotuzumab-induced ADCC did not correlate with the expression of SAP and EAT-2, small adaptor proteins that regulate signaling through SLAM family receptors, including SLAMF7. Interestingly, CLL cell lines that harbor the deletion of chromosome 17p displayed an increased expression of SLAMF7/CD319. This suggests that p53 dysregulation may influence *SLAMF7* gene regulation, resulting in the upregulation of its expression.

Most of the available literature on elotuzumab-induced ADCC focuses on multiple myeloma (MM) rather than CLL. SLAMF7/CD319 is highly expressed on MM cells. Several preclinical studies have clearly demonstrated that elotuzumab induces strong ADCC against MM cells in models using PBMCs or NK cells, with the effect being dependent on SLAMF7 receptor expression [37,38]. Beyond MM, elotuzumab has also been tested in B-cell malignancies, specifically plasmablastic lymphoma (PBL) and primary effusion lymphoma (PEL). In a study investigating PBL, the BC2 PBL cell line was used as a target, and SLAMF7/CD319 expression was shown to be comparable to that of MM cell lines (OPM). Using PBMCs as effector cells, this study demonstrated that elotuzumab induced significantly higher ADCC activity compared to controls after 4 h of incubation [12]. Another study examined the ability of elotuzumab to induce ADCC against PEL cells, a highly aggressive B-cell non-Hodgkin lymphoma. Seven PEL cell lines were used, all of which exhibited high SLAMF7/CD319 expression on their surface. Expanded NK cells immunomagnetically isolated from PBMCs were used as effector cells. After 4 h of incubation, elotuzumab induced high ADCC compared to the negative control, reaching 45–55% cytotoxicity [13]. These studies further support the concept that SLAMF7/CD319 may serve as a functional therapeutic target beyond MM.

Despite the lower expression of SLAMF7/CD319 on CLL cells compared to MM cells, elotuzumab may induce cytotoxicity through alternative mechanisms of action. Collins et al. (2013) were the first to provide in vitro evidence that elotuzumab can activate NK cells by direct binding to the SLAMF7/CD319 receptor on their surface [29]. This Fc-independent activation of NK cells suggests a mechanism involving the direct engagement of the SLAMF7/CD319 receptors via the Fab domains of elotuzumab. The findings of Collins et al. also suggest that elotuzumab may promote SLAMF7–SLAMF7 interactions between NK cells and myeloma cells, thereby enhancing natural cytotoxicity in addition to its well-established ability to promote ADCC [29]. This alternative mechanism may also explain why we were able to demonstrate elotuzumab-induced ADCC in CLL cell lines expressing SLAMF7/CD319 at levels lower than those of MM cells. Importantly, elotuzumab-induced ADCC was observed even in CLL cell lines with relatively low SLAMF7/CD319 expression among the tested lines. Specifically, in the CI, HG-3, and WA-OSEL cell lines, which exhibited lower expression of SLAMF7/CD319 compared to MEC-1 and MEC-2, measurable ADCC effects were detected. When NK cells were used as effector cells, ADCC levels reached 17.2 ± 8.6% in CI, 12.2 ± 8.5% in HG-3, and 8.8 ± 10.4% in WA-OSEL.

The intensity of ADCC induced by monoclonal antibodies may also depend on the type of effector cells used. When NK cells were used as effector cells, higher percentages of ADCC were observed for elotuzumab, rituximab, and their combination in nearly all cell lines, although the differences between ADCC induced by NK versus PBMCs-induced ADCC reached statistical significance only in the case of MEC-1 and CI cell lines. These findings are consistent with the results of Beum et al., 2008, who investigated which cellular populations within PBMCs primarily mediate ADCC [39]. Using the Daudi B-cell line as target cells, they demonstrated that NK cells are the main effector cells responsible for ADCC within the population of PBMCs. In the absence of NK cells, PBMCs exhibited only minimal ADCC activity [39]. These results explain why PBMCs may show lower ADCC activity compared to purified NK cells.

Contrary to our expectations, no synergism was observed between elotuzumab and rituximab. We hypothesized that elotuzumab might potentiate the ADCC effect of rituximab mainly by stimulating the activity of NK cells through binding to their SLAMF7/CD319 receptors. There are several possible explanations for the lack of a synergistic effect. Firstly, it was shown that a long 72 h pre-incubation of NK cells with elotuzumab is required to induce their activation [29]. In our study, both antibodies were added at the same time. Another possible mechanistic explanation could be that elotuzumab and rituximab compete for the same effector cells and FcγRIIIa receptors on NK cells. NK cells express limited FcγRIIIa, so simultaneous binding by elotuzumab and rituximab competes for engagement, reducing overall cytotoxicity rather than enhancing it.

## 4. Materials and Methods

### 4.1. Cell Lines and Cultures

In this study, six human CLL cell lines, MEC-1, MEC-2, CI, HG-3, PGA-1, and WA-OSEL, were used in this study. These cell lines were obtained from the Leibniz Institute, DSMZ–German Collection of Microorganisms and Cell Cultures (DSMZ, Braunschweig, Germany). MEC-1 and MEC-2 cells were cultured in Iscove’s Modified Dulbecco’s Medium (IMDM) (Biowest, Nuaillé, France) supplemented with 10% heat-inactivated fetal bovine serum (hiFBS) (Serana Europe, Pessin, Germany). The CI, HG-3, PGA-1, and WA-OSEL cell lines were maintained in RPMI 1640 medium (Biowest, Nuaillé, France). CI cells were cultured with 20% hiFBS, while HG-3, PGA-1, and WA-OSEL cells were cultured with 10% hiFBS. All culture media were supplemented with 1% antibiotic/antimycotic solution (Merck, Darmstadt, Germany). Cells were maintained under standard conditions at 37 °C in a humidified atmosphere containing 5% CO_2_. Before all experiments, cell viability was greater than 95% as determined by light microscopy (trypan blue staining) and flow cytometry (7-Aminoactinomycin D (7-AAD) staining).

### 4.2. Immunophenotypic Characterization of CLL Cell Lines by Flow Cytometry

Firstly, CLL cell lines were immunophenotypically characterized by 8-color 10-parameter flow cytometry using an adapted EuroFlow B-CLPD panel of antibodies [40]. The composition of the adapted B-CLPD panel and technical information on reagents are provided in Supplementary Table S1. Following immunophenotypic characterization, the expression of all nine SLAMF receptors as well as their intracellular adaptor proteins SAP (SLAM-associated protein) and EAT-2 (Ewing’s sarcoma-associated transcript 2) was analyzed in all cell lines using our own 8-color 10-parameter antibody panel (SLAMFR panel), as described previously [41]. The composition of the SLAMF panel and technical information on reagents are provided in Supplementary Table S2. The titers of all antibodies used were experimentally determined prior to the start of the study. For cell surface marker staining, the stain–wash–acquire method was used. For intracellular staining of SAP and EAT-2, the PerFix EXPOSE Kit (Beckman Coulter, Marseille, France) was used following the manufacturer’s instructions. After staining, the samples were washed, resuspended in PBS/BSA/EDTA solution, and acquired using a Navios EX flow cytometer (Beckman Coulter, Lismeehan, Ireland). Instrument setup and monitoring were performed using Flow-Set Pro and Flow-Check Pro fluorospheres (Beckman Coulter, Marseille, France), with target values defined by ClearLLab for the Navios EX cytometer. The acquired data were analyzed using the Infinicyt software package v.2.0.5.b.009 (Cytognos, Salamanca, Spain). Stain indices (SI) were calculated for all SLAMF receptors, SAP and EAT-2, using the following formula:

$$SI=\frac{MFI\;positive\;population-MFI\;negative\;population}{2\;*\;SD\;negative\;population}$$

### 4.3. Isolation of PBMCs

Peripheral blood samples were obtained from five healthy volunteers (2 female and 3 male donors) aged 25 to 52 years. Informed consent was obtained from all donors prior to sample collection. Peripheral blood mononuclear cells (PBMCs) were isolated from peripheral blood by density gradient centrifugation using Lymphocyte Separation Medium 1077 (LSM) (PromoCell, Heidelberg, Germany). Peripheral blood was layered onto LSM at a ratio of 1:1 and centrifuged at 440× *g* for 40 min at room temperature. The PBMC layer was collected and washed twice with PBS. The cells were resuspended in the culture medium, counted, and their viability and purity were determined by light microscopy (trypan blue staining) and flow cytometry (CD45/CD3/CD56/CD19/7-AAD staining). Cell viability was always greater than 95%, and more than 95% of PBMCs were lymphocytes. The isolated PBMCs were used directly for ADCC assays or for immunomagnetic isolation of NK cells.

### 4.4. Immunomagnetic Isolation of NK Cells

Natural killer (NK) cells were isolated from PBMCs using the Dynabeads™ Untouched™ Human NK Cells Kit (Invitrogen, Waltham, MA, USA), following the manufacturer’s instructions. Before use, Dynabeads were washed as recommended. PBMCs were resuspended in PBS, and 100 μL of hiFBS and 100 μL of antibody mix were added. The suspension was thoroughly mixed and incubated for 20 min at 4 °C. After incubation, the PBMCs were washed, mixed with Dynabeads, and incubated for 15 min at room temperature with gentle tilting and rotation. Following incubation, the cell suspension was gently mixed and placed in the Dynamag™-15 (Invitrogen, Waltham, MA, USA) magnet for 2 min. The supernatant, which contained the untouched human NK cells, was carefully transferred to a new tube. To remove any residual magnetic particles, the tube was placed on the magnet for an additional 2 min, and the supernatant containing the isolated NK cells was transferred to a fresh tube. The viability and purity of NK cells were determined by light microscopy (trypan blue staining) and flow cytometry (CD45/CD3/CD56/CD19/7-AAD staining). Cell viability was always greater than 95% and approximately 98% of the isolated cells were NK cells.

### 4.5. ADCC Analysis by Flow Cytometry

To evaluate ADCC induced by elotuzumab (Empliciti, Bristol-Myers Squibb Pharma, Dublin, Ireland) and rituximab (Ruxience, Pfizer Europe, Brussels, Belgium), an adapted flow cytometric method according to Yamasita et al. [27] was used. Human CLL cell lines (MEC-1, MEC-2, CI, HG-3, PGA-1, and WA-OSEL) were used as target cells, while PBMCs and NK cells served as effector cells. The target cells were labeled with carboxyfluorescein succinimidyl ester (CFSE) (BioLegend, San Diego, CA, USA). Target (T) cells were incubated with 1 µM CFSE for 20 min at room temperature, protected from light. After incubation, CFSE was neutralized with culture medium containing 10% hiFBS. Cells were washed, resuspended in culture medium, and seeded in 96-well culture plates. Effector (E) cells (PBMCs or NK cells) were added in a ratio of 8:1 (E:T) and co-incubated with target cells in the presence of monoclonal antibodies to initiate ADCC. Both elotuzumab and rituximab were used at final concentrations of 100 μg/mL. Negative control wells contained target cells only (to assess spontaneous/natural cell death), target cells with monoclonal antibodies but without effector cells (to assess cytotoxicity of antibodies alone), and target cells with effector cells but without monoclonal antibodies (to assess background/non-specific killing by effector cells themselves). The prepared plates were incubated for 4 h or 24 h under standard conditions (37 °C, humidified atmosphere with 5% CO_2_). Following incubation, cells were harvested and stained with 5 µL of 7-AAD (Beckman Coulter, Marseille, France) in 100 µL of cell suspension, which selectively labels dead cells. After 15 min of incubation, cells were washed with PBS and analyzed using a Navios EX flow cytometer (Beckman Coulter, Lismeehan, Ireland). The percentage of dead target cells was determined as the proportion of double-positive CFSE^+^/7-AAD^+^ cells out of total CFSE+ cells. The ADCC was calculated using the following formula:

% ADCC= % dead target cells (induced by effector cells in the presence of antibody)       −% dead cells(induced by effector cells in the absence of antibody)

### 4.6. Statistical Analysis

For statistical analyses, SPSS software version 31.0.1.0 (49) (IBM, Armonk, NY, USA) was used. The mean percentage of ADCC  ±  standard deviation (SD) was calculated from 3 to 6 replicate experiments. To establish the statistical significance of differences observed between groups, the non-parametric Mann–Whitney U-test was used. For comparison of paired samples, the paired samples *t*-test was used. For the analysis of correlations between two variables, the Spearman correlation coefficient was calculated. Statistical significance was considered to be present once *p* values were lower than 0.05.

## 5. Conclusions

Here, we found that elotuzumab, an anti-SLAMF7/CD319 antibody, induces significant ADCC against a panel of CLL cell lines, including MEC-1, MEC-2, CI, HG-3, PGA-1, and WA-OSEL. Generally, the effect of elotuzumab was slightly weaker than the effect of rituximab. Elotuzumab showed a more potent effect in MEC-1 cells, while rituximab was stronger in the remaining cell lines. CLL cell lines with higher SLAMF7/CD319 expression were more sensitive to elotuzumab-induced ADCC. Stronger SLAMF7/CD319 expression was observed in cell lines with del17p. The combination of elotuzumab with rituximab did not produce a significantly greater ADCC effect than either drug alone. In conclusion, our results suggest that elotuzumab is able to induce significant ADCC against CLL cells. Therefore, its potential as a possible therapy of CLL should be further evaluated.

## Figures and Tables

**Table 1:** ADCC induced by elotuzumab and rituximab in CLL cell lines.

	PBMC			NK Cells		
Cell Line	E	R	E + R	E	R	E + R
MEC-1	14.6 ± 8.1 *p* < 0.01	12.1 ± 7.2 *p* < 0.01	6.7 ± 5.4 *p* < 0.05	27.3 ± 4.7 *p* = 0.01 *p < 0.05*	22.5 ± 14.0 NS *NS*	26.8 ± 9.2 *p* < 0.05 *NS*
MEC-2	10.5 ± 7.0 *p* < 0.05	15.4 ± 9.1 *p* < 0.05	14.7 ± 12.2 NS	13.7 ± 5.5 *p* < 0.05 *NS*	22.6 ± 6.9 *p* < 0.01 *NS*	25.9 ± 12.9 *p* < 0.05 *NS*
CI	1.8 ± 2.2 NS	14.1 ± 8.9 *p* < 0.05	5.1 ± 2.0 *p* < 0.05	17.2 ± 8.6 *p* < 0.05 *p < 0.05*	27.5 ± 13.6 *p* < 0.05 *NS*	29.0 ± 16.2 *p* < 0.05 *p < 0.05*
HG-3	4.2 ± 2.5 *p* < 0.05	18.0 ± 6.9 *p* < 0.01	9.6 ± 3.9 *p* < 0.05	12.2 ± 8.5 NS *NS*	24.6 ± 7.2 *p* < 0.01 *NS*	24.2 ± 12.3 *p* < 0.05 *NS*
PGA-1	1.3 ± 1.2 NS	9.2 ± 4.6 *p* < 0.01	7.6 ± 3.9 *p* < 0.05	1.8 ± 3.7 NS *NS*	5.1 ± 4.3 NS *NS*	11.4 ± 9.5 NS *NS*
WA-OSEL	9.9 ± 2.7 *p* < 0.001	16.6 ± 9.4 *p* < 0.05	11.8 ± 7.0 *p* < 0.05	8.8 ± 10.4 NS *NS*	18.4 ± 5.1 *p* < 0.01 *NS*	16.4 ± 8.6 *p* < 0.05 *NS*

Results are expressed as mean % ADCC ± SD. E, elotuzumab; R, rituximab; E + R, the combination of elotuzumab with rituximab. Statistically significant differences in the number of dead cells between control (target cells + effector cells) vs. treatment (target cells + effector cells + antibody/ies): *p* < 0.001; *p* < 0.01; *p* < 0.05; NS, non-significant. Statistically significant differences in % ADCC induced by PBMC vs. NK cells (*in italics*): *p* < 0.05; *NS*, non-significant.

## Data Availability

The original contributions presented in this study are included in the article/Supplementary Materials. Further inquiries can be directed to the corresponding author.

## Supplementary Materials

The following supporting information can be downloaded at: https://www.mdpi.com/article/10.3390/molecules31030531/s1, Figure S1: Immunophenotypic characteristics of CLL cell lines: (a) MEC-1, (b) MEC-2, (c) CI, (d) HG-3, (e) PGA-1, (f) WA-OSEL.; Figure S2: The expression of SLAMF receptors, SAP and EAT-2 in CLL cell lines: (a) MEC-1, (b) MEC-2, (c) CI, (d) HG-3, (e) PGA-1, (f) WA-OSEL. Grey dots, control (unstained) cells; red dots, cells stained with the corresponding antibody.; Table S1: Composition of B-CLPD panel and technical information on reagents.; Table S2: Composition of SLAMFR panel and technical information on reagents.

## Abbreviations

The following abbreviations are used in this manuscript:

7-AAD	7-amino-actinomycin D
ADCC	antibody-dependent cellular cytotoxicity
CFSE	carboxyfluorescein succinimidyl ester
CLL	chronic lymphocytic leukemia
E	elotuzumab
EAT-2	Ewing’s sarcoma-Associated Transcript 2
hiFBS	heat-inactivated fetal bovine serum
MM	multiple myeloma
PBMCs	peripheral blood mononuclear cells
PBL	plasmablastic lymphoma
PEL	primary effusion lymphoma
R	rituximab
SAP	SLAM-associated protein
SLAMFR	signaling lymphocytic activation molecule family receptor
