# Supplementary material for: The Anti-SLAMF7 Antibody, Elotuzumab, Induces Antibody-Dependent Cellular Cytotoxicity Against CLL Cell Lines
Source: Molecules. 2026 Feb 3;31(3):531. doi: 10.3390/molecules31030531 (PMC12899419; doi:10.3390/molecules31030531)
Supplement: Supplementary file 1 [file molecules-31-00531-s001.zip › Supplementary Figure S1.pdf]

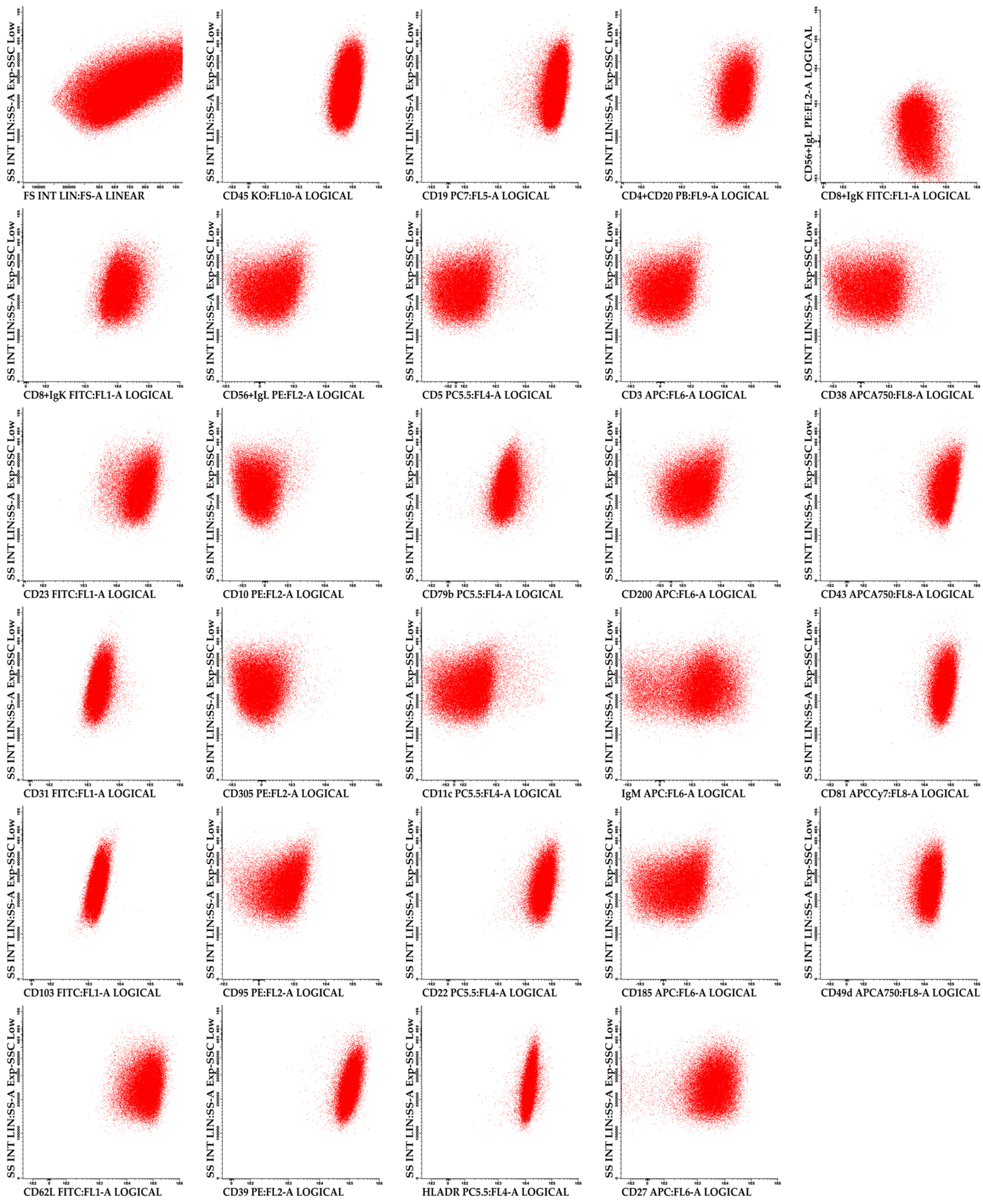

(a) MEC-1

Supplementary Figure S1. Immunophenotypic characteristics of CLL cell lines: (a) MEC-1, (b) MEC-2, (c) CI, (d) HG-3, (e) PGA-1, (f) WA-OSEL.

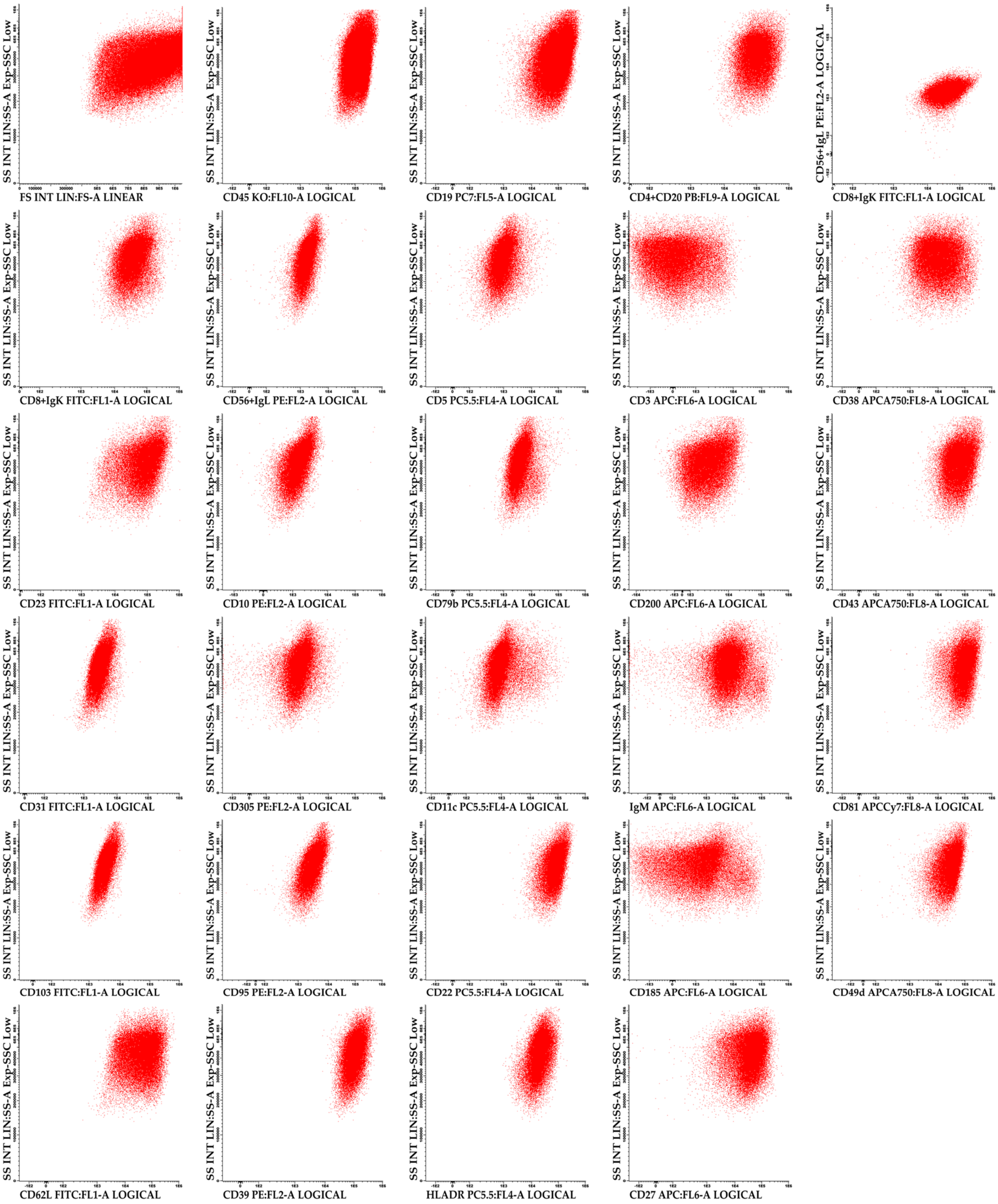
SS INT LIN:SS-A Exp-SSC Low
FS INT LIN:FS-A LINEAR
CD45 KO:FL10-A LOGICAL
CD19 PC7:FL5-A LOGICAL
CD4+CD20 PB:FL9-A LOGICAL
CD56+IgL PE:FL2-A LOGICAL
CD8+IgK FITC:FL1-A LOGICAL
CD8+IgK FITC:FL1-A LOGICAL
CD56+IgL PE:FL2-A LOGICAL
CD5 PC5.5:FL4-A LOGICAL
CD3 APC:FL6-A LOGICAL
CD38 APCA750:FL8-A LOGICAL
CD23 FITC:FL1-A LOGICAL
CD10 PE:FL2-A LOGICAL
CD79b PC5.5:FL4-A LOGICAL
CD200 APC:FL6-A LOGICAL
CD43 APCA750:FL8-A LOGICAL
CD31 FITC:FL1-A LOGICAL
CD305 PE:FL2-A LOGICAL
CD11c PC5.5:FL4-A LOGICAL
IgM APC:FL6-A LOGICAL
CD81 APCCy7:FL8-A LOGICAL
CD103 FITC:FL1-A LOGICAL
CD95 PE:FL2-A LOGICAL
CD22 PC5.5:FL4-A LOGICAL
CD185 APC:FL6-A LOGICAL
CD49d APCA750:FL8-A LOGICAL
CD62L FITC:FL1-A LOGICAL
CD39 PE:FL2-A LOGICAL
HLADR PC5.5:FL4-A LOGICAL
CD27 APC:FL6-A LOGICAL

(b) MEC-2

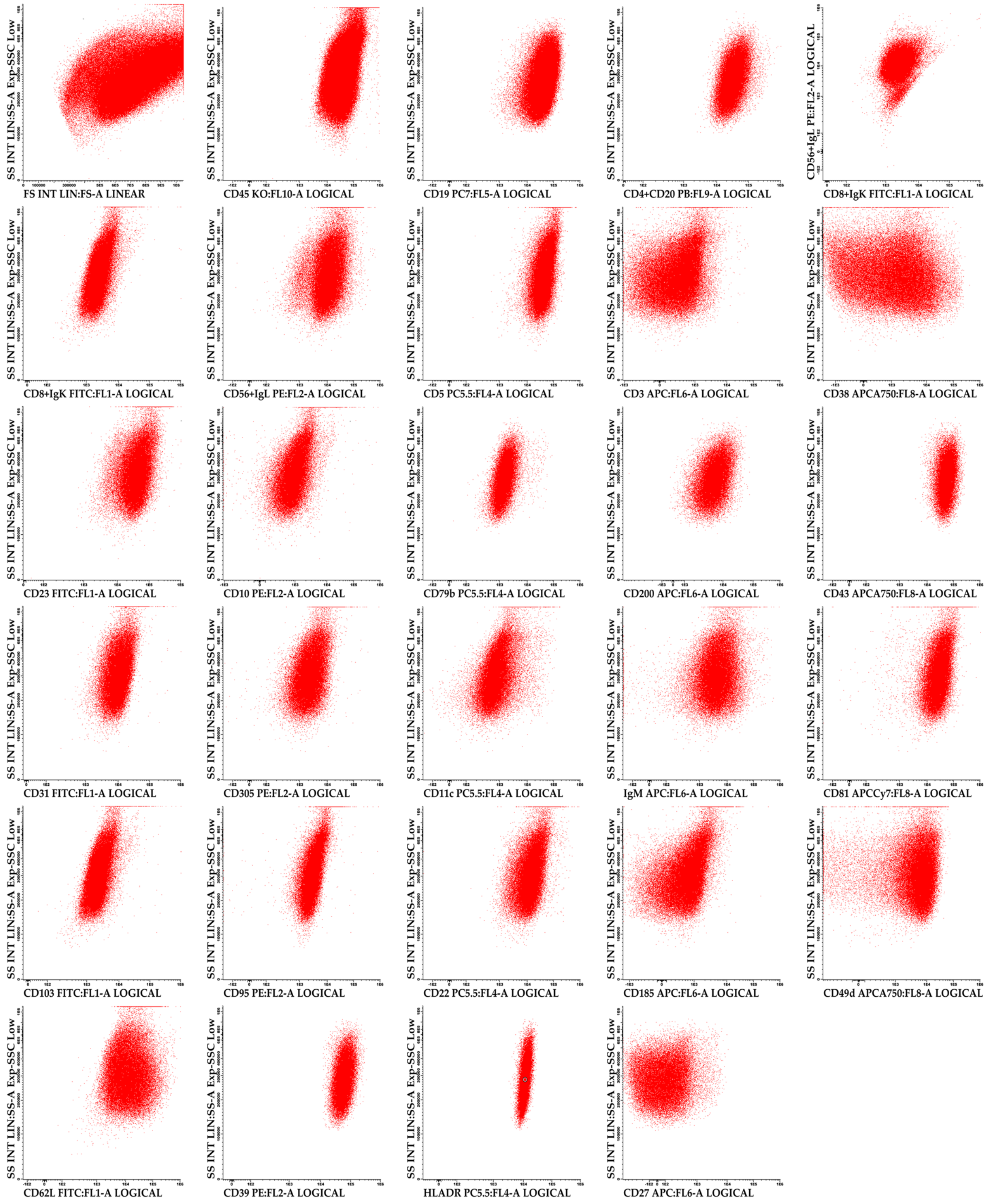
SS INT LIN:SS-A Exp-SSC Low
FS INT LIN:FS-A LINEAR
CD45 KO:FL10-A LOGICAL
CD19 PC7:FL5-A LOGICAL
CD4+CD20 PB:FL9-A LOGICAL
CD56+IgL PE:FL2-A LOGICAL
CD8+IgK FITC:FL1-A LOGICAL
CD8+IgK FITC:FL1-A LOGICAL
CD56+IgL PE:FL2-A LOGICAL
CD5 PC5.5:FL4-A LOGICAL
CD3 APC:FL6-A LOGICAL
CD38 APCA750:FL8-A LOGICAL
CD23 FITC:FL1-A LOGICAL
CD10 PE:FL2-A LOGICAL
CD79b PC5.5:FL4-A LOGICAL
CD200 APC:FL6-A LOGICAL
CD43 APCA750:FL8-A LOGICAL
CD31 FITC:FL1-A LOGICAL
CD305 PE:FL2-A LOGICAL
CD11c PC5.5:FL4-A LOGICAL
IgM APC:FL6-A LOGICAL
CD81 APCCy7:FL8-A LOGICAL
CD103 FITC:FL1-A LOGICAL
CD95 PE:FL2-A LOGICAL
CD22 PC5.5:FL4-A LOGICAL
CD185 APC:FL6-A LOGICAL
CD49d APCA750:FL8-A LOGICAL
CD62L FITC:FL1-A LOGICAL
CD39 PE:FL2-A LOGICAL
HLADR PC5.5:FL4-A LOGICAL
CD27 APC:FL6-A LOGICAL

(c) CI

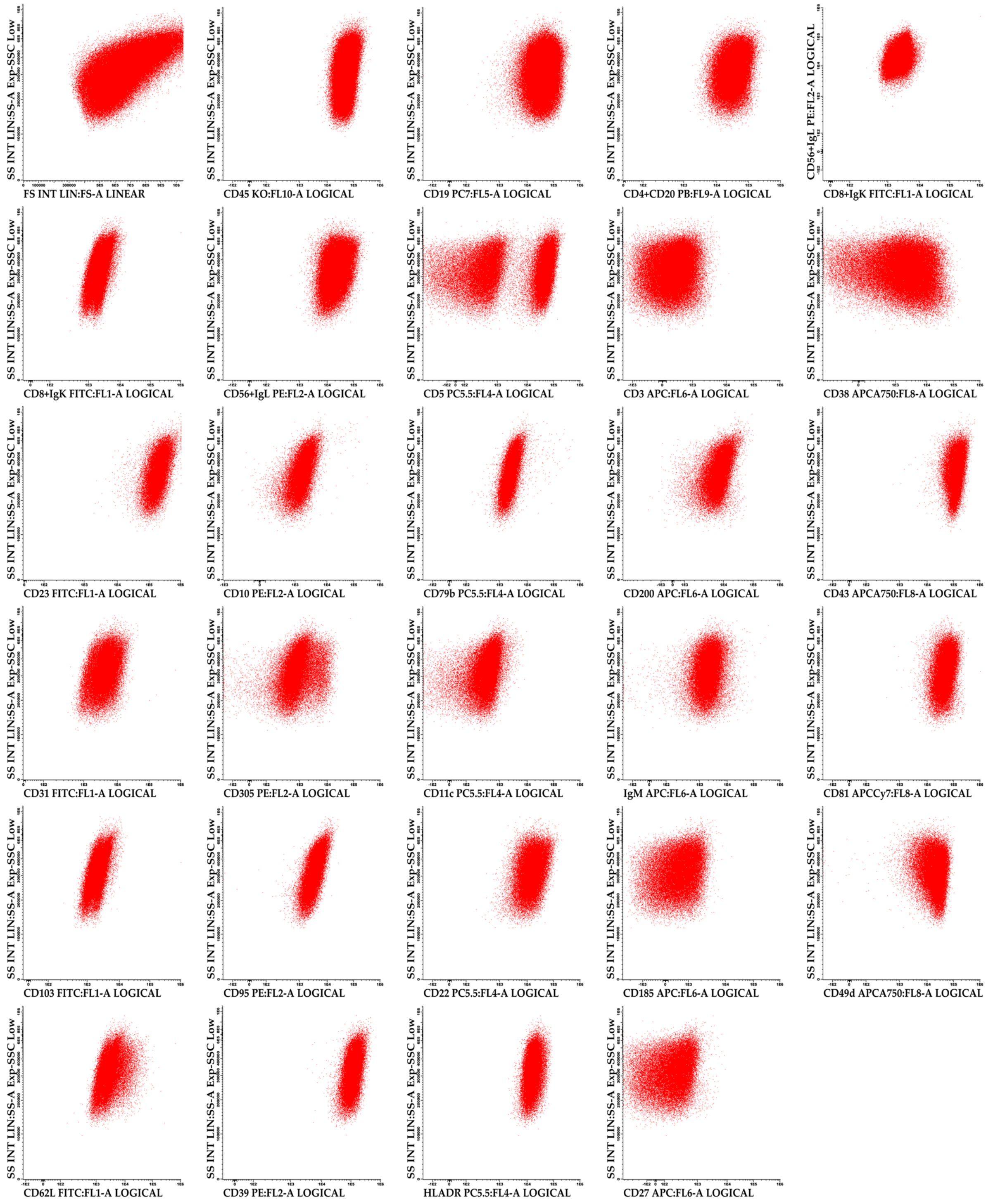
SS INT LIN:SS-A Exp-SSC Low
FS INT LIN:FS-A LINEAR
CD45 KO:FL10-A LOGICAL
CD19 PC7:FL5-A LOGICAL
CD4+CD20 PB:FL9-A LOGICAL
CD56+IgL PE:FL2-A LOGICAL
CD8+IgK FITC:FL1-A LOGICAL
CD8+IgK FITC:FL1-A LOGICAL
CD56+IgL PE:FL2-A LOGICAL
CD5 PC5.5:FL4-A LOGICAL
CD3 APC:FL6-A LOGICAL
CD38 APCA750:FL8-A LOGICAL
CD23 FITC:FL1-A LOGICAL
CD10 PE:FL2-A LOGICAL
CD79b PC5.5:FL4-A LOGICAL
CD200 APC:FL6-A LOGICAL
CD43 APCA750:FL8-A LOGICAL
CD31 FITC:FL1-A LOGICAL
CD305 PE:FL2-A LOGICAL
CD11c PC5.5:FL4-A LOGICAL
IgM APC:FL6-A LOGICAL
CD81 APCCy7:FL8-A LOGICAL
CD103 FITC:FL1-A LOGICAL
CD95 PE:FL2-A LOGICAL
CD22 PC5.5:FL4-A LOGICAL
CD185 APC:FL6-A LOGICAL
CD49d APCA750:FL8-A LOGICAL
CD62L FITC:FL1-A LOGICAL
CD39 PE:FL2-A LOGICAL
HLADR PC5.5:FL4-A LOGICAL
CD27 APC:FL6-A LOGICAL

(d) HG-3

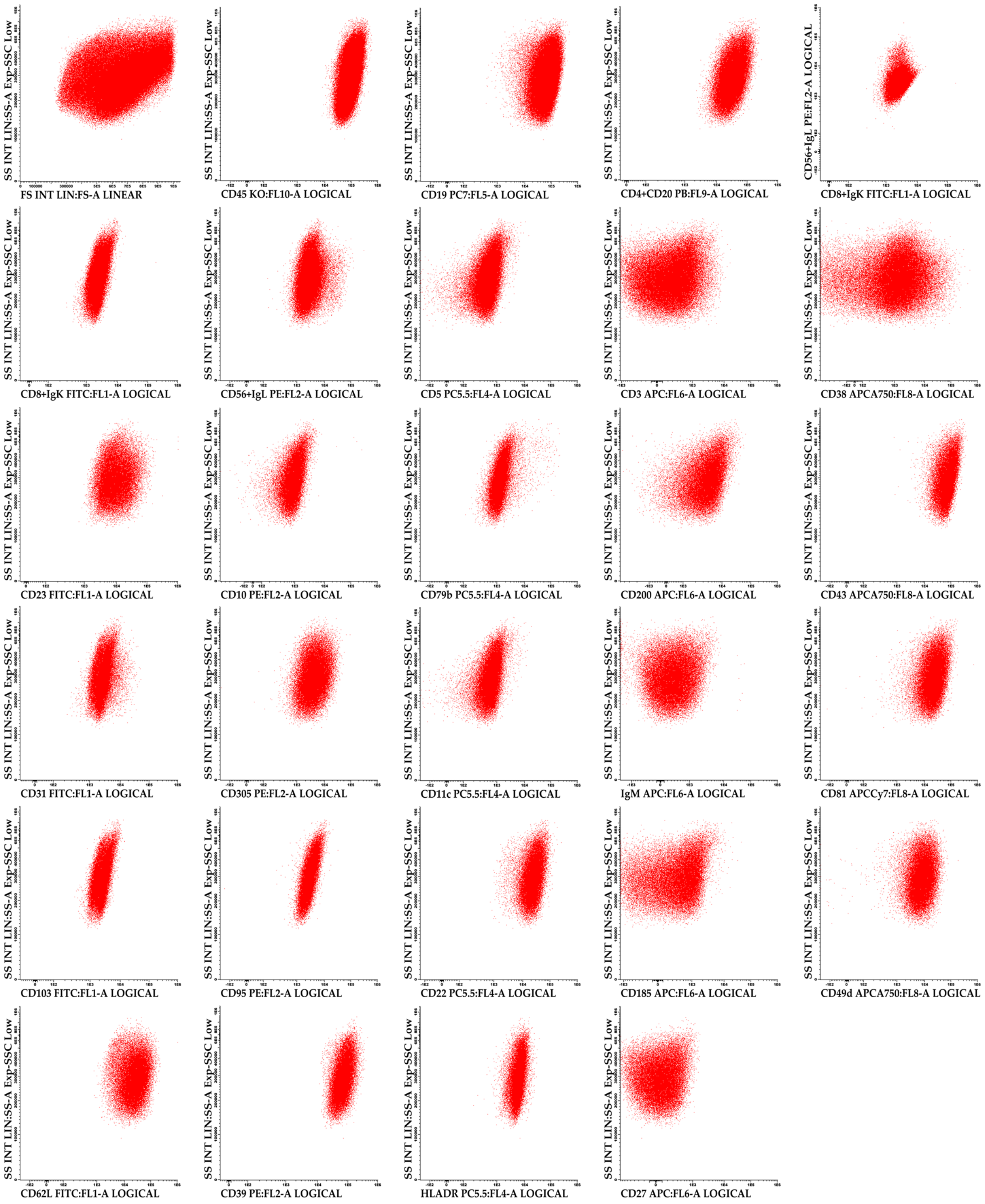
SS INT LIN:SS-A Exp-SSC Low
FS INT LIN:FS-A LINEAR
CD45 KO:FL10-A LOGICAL
CD19 PC7:FL5-A LOGICAL
CD4+CD20 PB:FL9-A LOGICAL
CD56+IgL PE:FL2-A LOGICAL
CD8+IgK FITC:FL1-A LOGICAL
CD8+IgK FITC:FL1-A LOGICAL
CD56+IgL PE:FL2-A LOGICAL
CD5 PC5.5:FL4-A LOGICAL
CD3 APC:FL6-A LOGICAL
CD38 APCA750:FL8-A LOGICAL
CD23 FITC:FL1-A LOGICAL
CD10 PE:FL2-A LOGICAL
CD79b PC5.5:FL4-A LOGICAL
CD200 APC:FL6-A LOGICAL
CD43 APCA750:FL8-A LOGICAL
CD31 FITC:FL1-A LOGICAL
CD305 PE:FL2-A LOGICAL
CD11c PC5.5:FL4-A LOGICAL
IgM APC:FL6-A LOGICAL
CD81 APCCy7:FL8-A LOGICAL
CD103 FITC:FL1-A LOGICAL
CD95 PE:FL2-A LOGICAL
CD22 PC5.5:FL4-A LOGICAL
CD185 APC:FL6-A LOGICAL
CD49d APCA750:FL8-A LOGICAL
CD62L FITC:FL1-A LOGICAL
CD39 PE:FL2-A LOGICAL
HLADR PC5.5:FL4-A LOGICAL
CD27 APC:FL6-A LOGICAL

(e) PGA-1

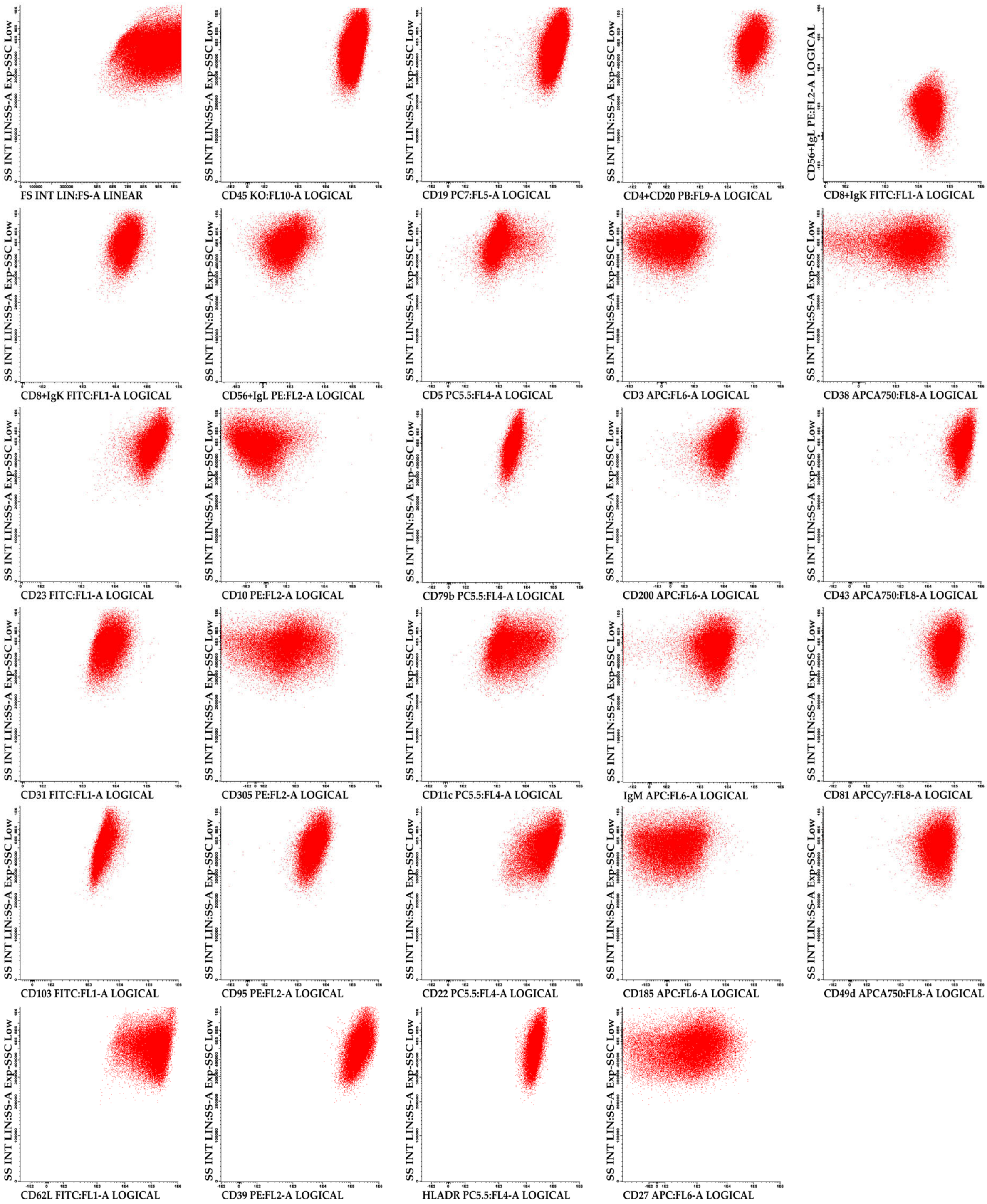
SS INT LIN:SS-A Exp-SSC Low
FS INT LIN:FS-A LINEAR
CD45 KO:FL10-A LOGICAL
CD19 PC7:FL5-A LOGICAL
CD4+CD20 PB:FL9-A LOGICAL
CD56+IgL PE:FL2-A LOGICAL
CD8+IgK FITC:FL1-A LOGICAL
CD8+IgK FITC:FL1-A LOGICAL
CD56+IgL PE:FL2-A LOGICAL
CD5 PC5.5:FL4-A LOGICAL
CD3 APC:FL6-A LOGICAL
CD38 APCA750:FL8-A LOGICAL
CD23 FITC:FL1-A LOGICAL
CD10 PE:FL2-A LOGICAL
CD79b PC5.5:FL4-A LOGICAL
CD200 APC:FL6-A LOGICAL
CD43 APCA750:FL8-A LOGICAL
CD31 FITC:FL1-A LOGICAL
CD305 PE:FL2-A LOGICAL
CD11c PC5.5:FL4-A LOGICAL
IgM APC:FL6-A LOGICAL
CD81 APCCy7:FL8-A LOGICAL
CD103 FITC:FL1-A LOGICAL
CD95 PE:FL2-A LOGICAL
CD22 PC5.5:FL4-A LOGICAL
CD185 APC:FL6-A LOGICAL
CD49d APCA750:FL8-A LOGICAL
CD62L FITC:FL1-A LOGICAL
CD39 PE:FL2-A LOGICAL
HLADR PC5.5:FL4-A LOGICAL
CD27 APC:FL6-A LOGICAL

(f) WA-OSEL
