# Supplementary material for: The Anti-SLAMF7 Antibody, Elotuzumab, Induces Antibody-Dependent Cellular Cytotoxicity Against CLL Cell Lines
Source: Molecules. 2026 Feb 3;31(3):531. doi: 10.3390/molecules31030531 (PMC12899419; doi:10.3390/molecules31030531)
Supplement: Supplementary file 1 [file molecules-31-00531-s001.zip › Supplementary Figure S2.pdf]

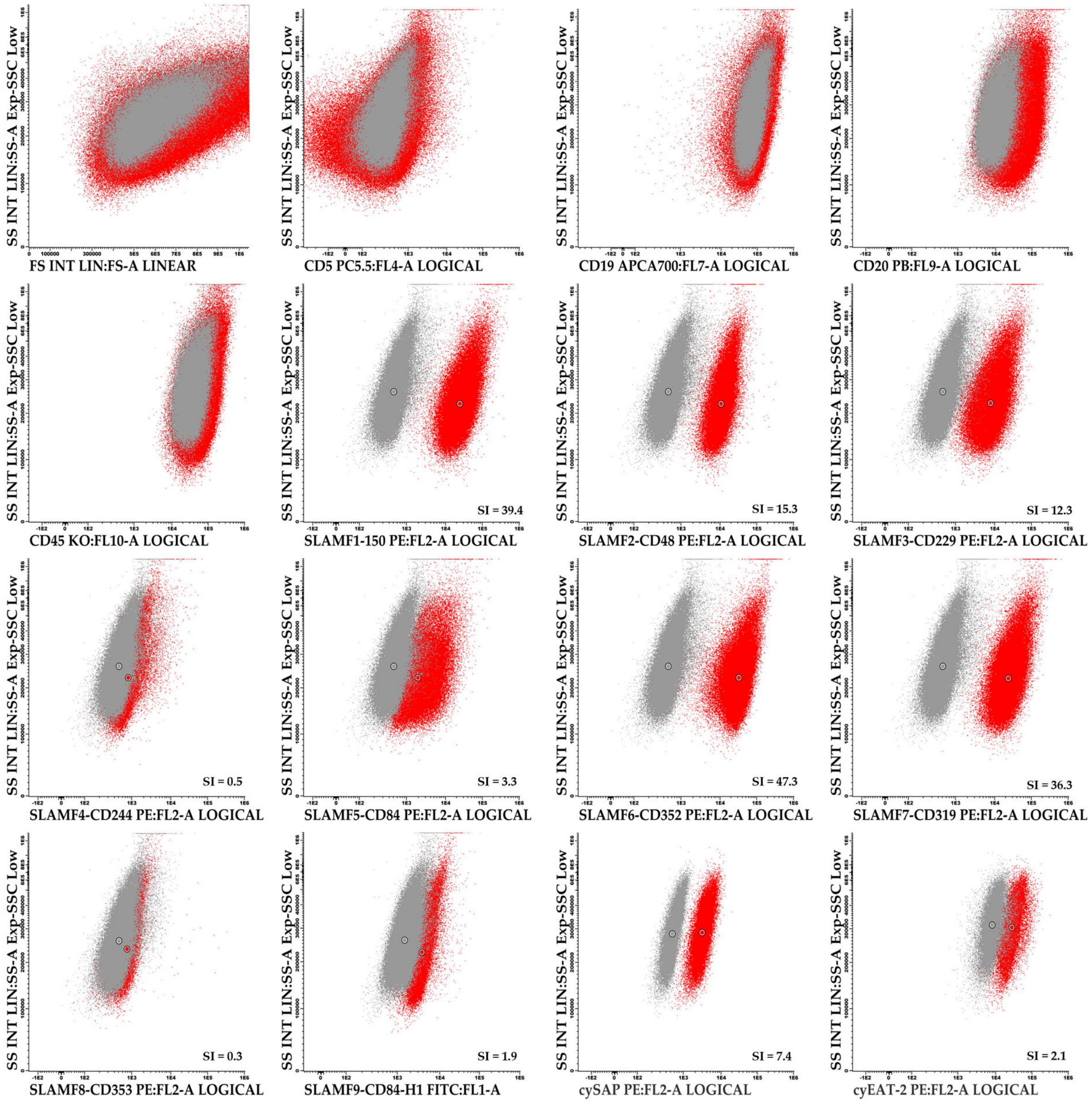

(a) MEC-1

Supplementary Figure S2. The expression of SLAMF receptors, SAP and EAT-2 in CLL cell lines: (a) MEC-1, (b) MEC-2, (c) CI, (d) HG-3, (e) PGA-1, (f) WA-OSEL. Grey dots, control (unstained) cells; red dots, cells stained with the corresponding antibody.

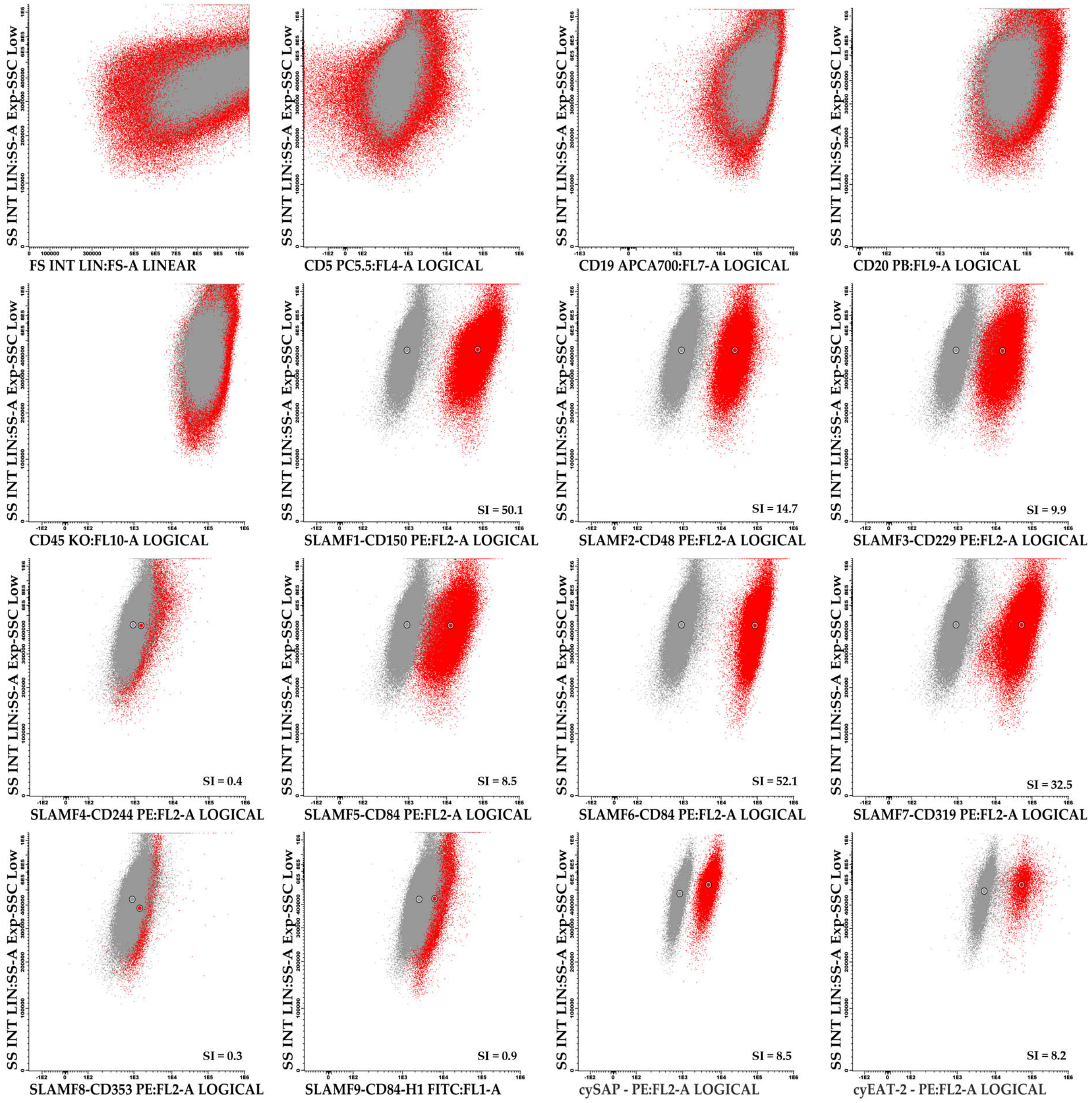
SS INT LIN:SS-A Exp-SSC Low
FS INT LIN:FS-A LINEAR
CD5 PC5.5:FL4-A LOGICAL
CD19 APCA700:FL7-A LOGICAL
CD20 PB:FL9-A LOGICAL
CD45 KO:FL10-A LOGICAL
SI = 50.1
SLAMF1-CD150 PE:FL2-A LOGICAL
SI = 14.7
SLAMF2-CD48 PE:FL2-A LOGICAL
SI = 9.9
SLAMF3-CD229 PE:FL2-A LOGICAL
SI = 0.4
SLAMF4-CD244 PE:FL2-A LOGICAL
SI = 8.5
SLAMF5-CD84 PE:FL2-A LOGICAL
SI = 52.1
SLAMF6-CD84 PE:FL2-A LOGICAL
SI = 32.5
SLAMF7-CD319 PE:FL2-A LOGICAL
SI = 0.3
SLAMF8-CD353 PE:FL2-A LOGICAL
SI = 0.9
SLAMF9-CD84-H1 FITC:FL1-A
SI = 8.5
cySAP - PE:FL2-A LOGICAL
SI = 8.2
cyEAT-2 - PE:FL2-A LOGICAL

(b) MEC-2

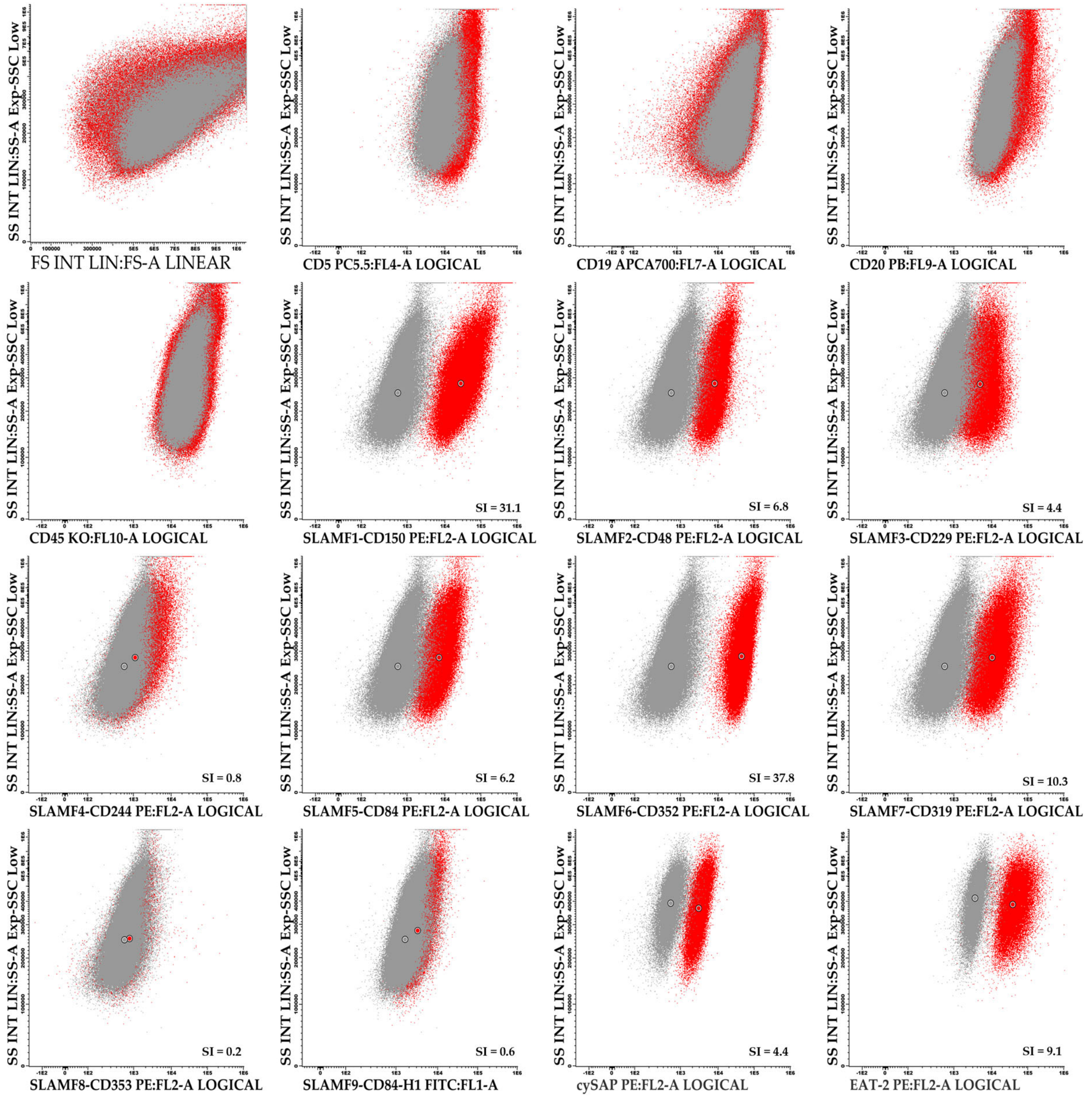
SS INT LIN:SS-A Exp-SSC Low
FS INT LIN:FS-A LINEAR
CD5 PC5.5:FL4-A LOGICAL
CD19 APCA700:FL7-A LOGICAL
CD20 PB:FL9-A LOGICAL
CD45 KO:FL10-A LOGICAL
SI = 31.1
SLAMF1-CD150 PE:FL2-A LOGICAL
SI = 6.8
SLAMF2-CD48 PE:FL2-A LOGICAL
SI = 4.4
SLAMF3-CD229 PE:FL2-A LOGICAL
SI = 0.8
SLAMF4-CD244 PE:FL2-A LOGICAL
SI = 6.2
SLAMF5-CD84 PE:FL2-A LOGICAL
SI = 37.8
SLAMF6-CD352 PE:FL2-A LOGICAL
SI = 10.3
SLAMF7-CD319 PE:FL2-A LOGICAL
SI = 0.2
SLAMF8-CD353 PE:FL2-A LOGICAL
SI = 0.6
SLAMF9-CD84-H1 FITC:FL1-A
SI = 4.4
cySAP PE:FL2-A LOGICAL
SI = 9.1
EAT-2 PE:FL2-A LOGICAL

(c) CI

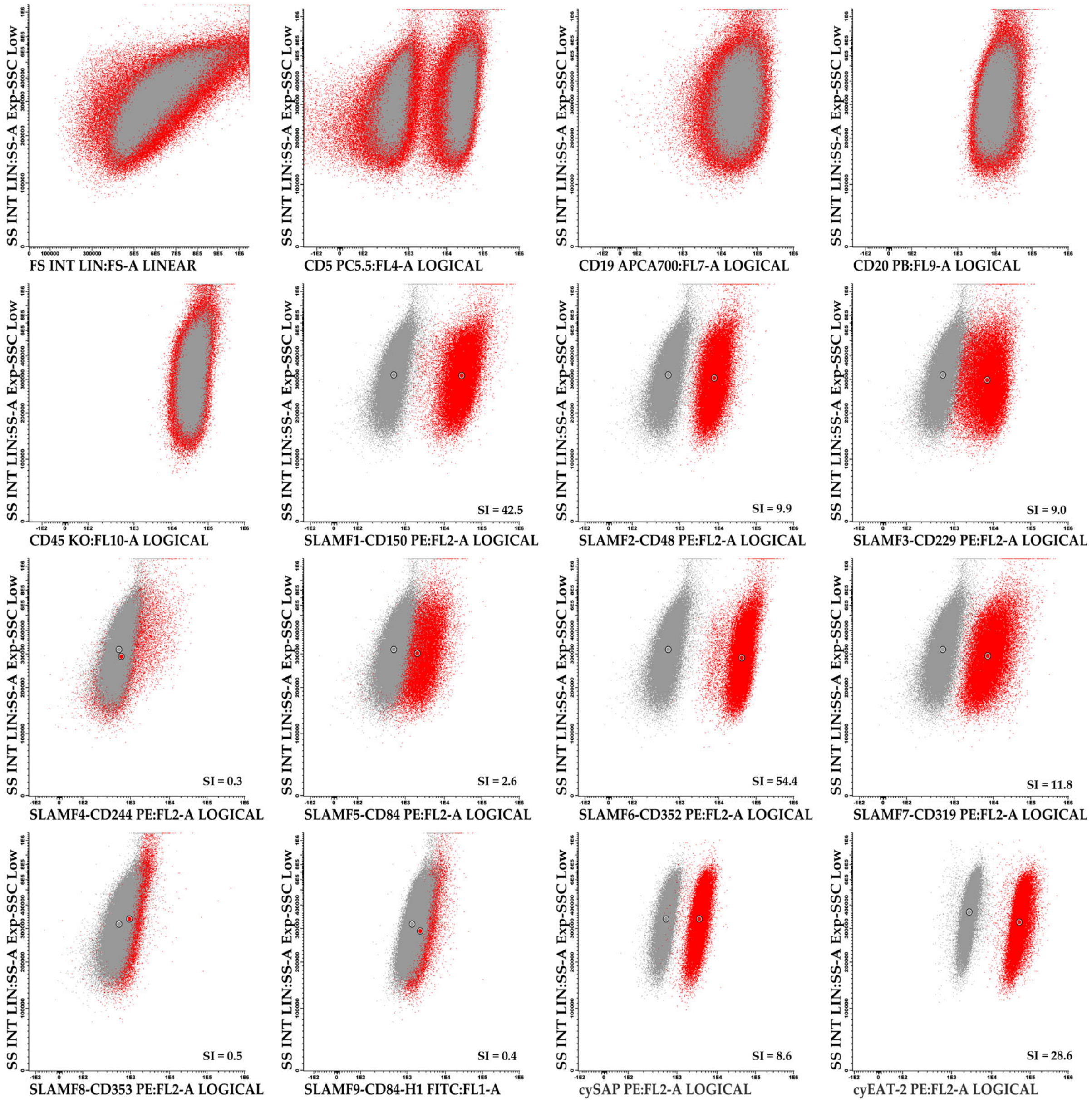
SS INT LIN:SS-A Exp-SSC Low
FS INT LIN:FS-A LINEAR
CD5 PC5.5:FL4-A LOGICAL
CD19 APCA700:FL7-A LOGICAL
CD20 PB:FL9-A LOGICAL
CD45 KO:FL10-A LOGICAL
SI = 42.5
SLAMF1-CD150 PE:FL2-A LOGICAL
SI = 9.9
SLAMF2-CD48 PE:FL2-A LOGICAL
SI = 9.0
SLAMF3-CD229 PE:FL2-A LOGICAL
SI = 0.3
SLAMF4-CD244 PE:FL2-A LOGICAL
SI = 2.6
SLAMF5-CD84 PE:FL2-A LOGICAL
SI = 54.4
SLAMF6-CD352 PE:FL2-A LOGICAL
SI = 11.8
SLAMF7-CD319 PE:FL2-A LOGICAL
SI = 0.5
SLAMF8-CD353 PE:FL2-A LOGICAL
SI = 0.4
SLAMF9-CD84-H1 FITC:FL1-A
SI = 8.6
cySAP PE:FL2-A LOGICAL
SI = 28.6
cyEAT-2 PE:FL2-A LOGICAL

(d) HG-3

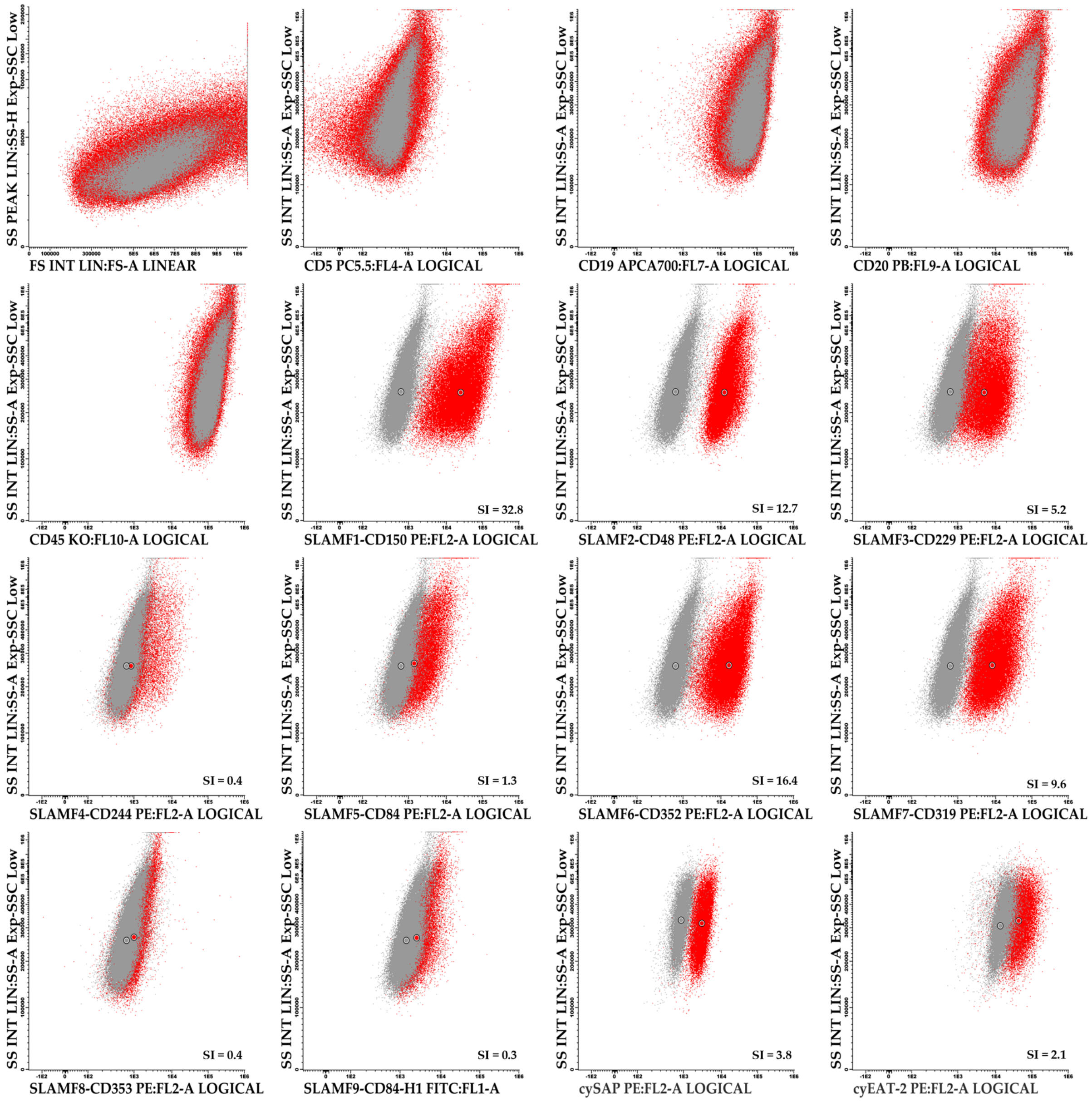
SS PEAK LIN:SS-H Exp-SSC Low
FS INT LIN:FS-A LINEAR
SS INT LIN:SS-A Exp-SSC Low
CD5 PC5.5:FL4-A LOGICAL
CD19 APCA700:FL7-A LOGICAL
CD20 PB:FL9-A LOGICAL
CD45 KO:FL10-A LOGICAL
SLAMF1-CD150 PE:FL2-A LOGICAL
SI = 32.8
SLAMF2-CD48 PE:FL2-A LOGICAL
SI = 12.7
SLAMF3-CD229 PE:FL2-A LOGICAL
SI = 5.2
SLAMF4-CD244 PE:FL2-A LOGICAL
SI = 0.4
SLAMF5-CD84 PE:FL2-A LOGICAL
SI = 1.3
SLAMF6-CD352 PE:FL2-A LOGICAL
SI = 16.4
SLAMF7-CD319 PE:FL2-A LOGICAL
SI = 9.6
SLAMF8-CD353 PE:FL2-A LOGICAL
SI = 0.4
SLAMF9-CD84-H1 FITC:FL1-A
SI = 0.3
cySAP PE:FL2-A LOGICAL
SI = 3.8
cyEAT-2 PE:FL2-A LOGICAL
SI = 2.1

(e) PGA-1

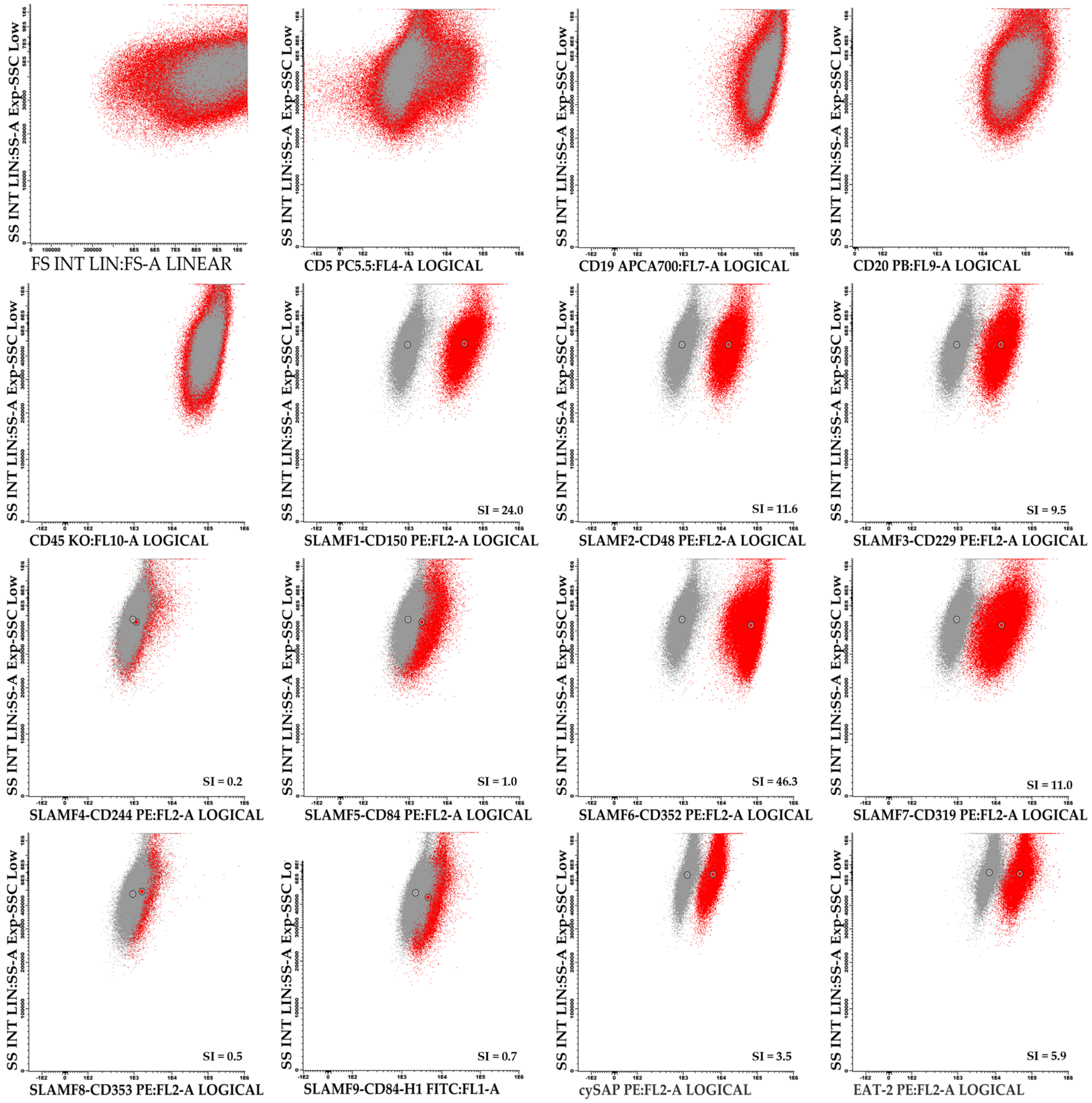

SS INT LIN:SS-A Exp-SSC Low
FS INT LIN:FS-A LINEAR
CD5 PC5.5:FL4-A LOGICAL
CD19 APCA700:FL7-A LOGICAL
CD20 PB:FL9-A LOGICAL
CD45 KO:FL10-A LOGICAL
SI = 24.0
SLAMF1-CD150 PE:FL2-A LOGICAL
SI = 11.6
SLAMF2-CD48 PE:FL2-A LOGICAL
SI = 9.5
SLAMF3-CD229 PE:FL2-A LOGICAL
SI = 0.2
SLAMF4-CD244 PE:FL2-A LOGICAL
SI = 1.0
SLAMF5-CD84 PE:FL2-A LOGICAL
SI = 46.3
SLAMF6-CD352 PE:FL2-A LOGICAL
SI = 11.0
SLAMF7-CD319 PE:FL2-A LOGICAL
SI = 0.5
SLAMF8-CD353 PE:FL2-A LOGICAL
SI = 0.7
SLAMF9-CD84-H1 FITC:FL1-A
SI = 3.5
cySAP PE:FL2-A LOGICAL
SI = 5.9
EAT-2 PE:FL2-A LOGICAL

(f) WA-OSEL
