# Supplementary material for: The Anti-SLAMF7 Antibody, Elotuzumab, Induces Antibody-Dependent Cellular Cytotoxicity Against CLL Cell Lines
Source: Molecules. 2026 Feb 3;31(3):531. doi: 10.3390/molecules31030531 (PMC12899419; doi:10.3390/molecules31030531)
Supplement: Supplementary file 1 [file molecules-31-00531-s001.zip › Supplementary Table S1_B-CLPD panel.pdf]

Supplementary Table S1. Composition of B-CLPD panel and technical information on reagents.

Tube	**PB**	**KO**	**FITC**	**PE**	**PC5.5**	**PC7**	**APC**	**APC-A750/-Cy7**
1	CD4+CD20	CD45	CD8+Igλ	CD56+Igκ	CD5	CD19+TCRγδ	CD3	CD38
2	CD20	CD45	CD23	CD10	CD79b	CD19	CD200	CD43
3	CD20	CD45	CD31	CD305	CD11c	CD19	IgM	CD81
4	CD20	CD45	CD103	CD95	CD22	CD19	CD185	CD49d
5	CD20	CD45	CD62L	CD39	HLADR	CD19	CD27	

**Antigen**	**Fluorochrome**	**Clone**	**Isotype**	**Manufacturer**
CD3	APC	UCHT1	mIgG1	Beckman Coulter
CD4	Pacific Blue	13B8.2	mIgG1	Beckman Coulter
CD5	PE-Cy5.5	BL1a	mIgG2a	Beckman Coulter
CD8	FITC	B9.11	mIgG1	Beckman Coulter
CD10	PE	ALB1	mIgG1	Beckman Coulter
CD11c	PE-Cy5-5	BU15	mIgG1	Beckman Coulter
CD19	PE-Cy7	J3-119	mIgG1	Beckman Coulter
CD20	Pacific Blue	B9E9	mIgG2a	Beckman Coulter
CD22	PE-Cy5.5	SJ10.1H11	mIgG1	Beckman Coulter
CD23	FITC	9P25	mIgG1	Beckman Coulter
CD27	APC	1A427	mIgG1	Beckman Coulter
CD32	FITC	5.6E	mIgG1	Beckman Coulter
CD38	APC-Alexa750	LS198-4-3	mIgG1	Beckman Coulter
CD39	PE	BA54	mIgG1	Beckman Coulter
CD43	APC-Alexa750	DFT1	mIgG1	Beckman Coulter
CD45	Krome Orange	J33	mIgG1	Beckman Coulter
CD49d	APC-Alexa750	HP2/1	mIgG1	Beckman Coulter
CD56	PE	N901	mIgG1	Beckman Coulter
CD62L	FITC	DREG56	mIgG1	Beckman Coulter
CD79b	PE-Cy5.5	CB3-1	mIgG1	Beckman Coulter
CD81	APC-Cy7	M38	mIgG1	EXBIO
CD95	PE	UB2	mIgG1	Beckman Coulter
CD103	FITC	2G5	mIgG2a	Beckman Coulter
CD185	APC	J252D4	mIgG1	BioLegend
CD200	APC	OX-104	mIgG1	EXBIO
CD305	PE	NKTA255	mIgG1	Beckman Coulter
IgKappa	FITC	polyclonal	F(ab′)2 goat	Beckman Coulter
IgLambda	PE	polyclonal	F(ab′)2 goat	Beckman Coulter
HLA-DR	PE-Cy5.5	Immu-357	mIgG1	Beckman Coulter
TCRγ/δ	PE-Cy7	IMMU510	mIgG1	Beckman Coulter
IgM	APC	SA-DA4	mIgG1	Beckman Coulter
