# Supplementary material for: The Anti-SLAMF7 Antibody, Elotuzumab, Induces Antibody-Dependent Cellular Cytotoxicity Against CLL Cell Lines
Source: Molecules. 2026 Feb 3;31(3):531. doi: 10.3390/molecules31030531 (PMC12899419; doi:10.3390/molecules31030531)
Supplement: Supplementary file 1 [file molecules-31-00531-s001.zip › Supplementary Table S2_SLAMFR panel.pdf]

Supplementary Table S2. Composition of SLAMFR panel and technical information on reagents.

Tube	PB	KO	FITC	PE	ECD	PC5.5	PC7	APC	APC-A700	APC-A750
1	CD4+CD20	CD45	mCTRL	mCTRL	-	CD5	CD10	CD3	CD19	CD8
2-9	CD4+CD20	CD45	-	SLAMF1-8	-	CD5	CD10	CD3	CD19	CD8
10	CD4+CD20	CD45	SLAMF9	-	-	CD5	CD10	CD3	CD19	CD8
11	CD4+CD20	CD45	cyCTRL	cyCTRL	-	CD5	CD10	CD3	CD19	CD8
12	CD4+CD20	CD45	-	cySAP	-	CD5	CD10	CD3	CD19	CD8
13	CD4+CD20	CD45	cyCTRL	cyCTRL +sec. Ab	-	CD5	CD10	CD3	CD19	CD8
14	CD4+CD20	CD45	-	cyEAT-2 +sec. Ab	-	CD5	CD10	CD3	CD19	CD8

Antigen	Fluorochrome	Clone	Isotype	Manufacturer
CD3	APC	UCHT1	mIgG1	Beckman Coulter
CD4	Pacific Blue	13B8.2	mIgG1	Beckman Coulter
CD5	PE-Cy5.5	BL.1a	mIgG2a	Beckman Coulter
CD8	APC-Alexa750	B9.11	mIgG1	Beckman Coulter
CD10	PE-Cy7	ALB1	mIgG1	Beckman Coulter
CD19	APC-Alexa700	J3-119	mIgG1	Beckman Coulter
CD20	Pacific Blue	B9E9	mIgG2a	Beckman Coulter
CD45	Krome Orange	J33	mIgG1	Beckman Coulter
SLAMF1/CD150	PE	SLAM.4	mIgG1	EXBIO
SLAMF2/CD48	PE	MEM-102	mIgG1	EXBIO
SLAMF3CD229	PE	Hly9.25	mIgG1	EXBIO
SLAMF4/CD244	PE	C1.7	mIgG1	BioLegend
SLAMF5/CD84	PE	CD84.1.21	mIgG2a	BioLegend
SLAMF6/CD352	PE	NT-7	mIgG1	BioLegend
SLAMF7/CD319	PE	162.1	mIgG2b	BioLegend
SLAMF8/CD353	PE	REA394/250014	recombinant huIgG1	MiltenyiBiotec
SLAMF9/CD84-H1	FITC	polyclonal	rbIgG	MyBioSource
SAP	PE	XLP-1D12	rIgG2a	Invitrogen
EAT-2	PE	polyclonal	rbIgG	Proteintech
Secondary anti-rabbit polyclonal antibody	PE	Poly4064	dyIgG	BioLegend
